# Transferrin receptor in primary and metastatic breast cancer: Evaluation of expression and experimental modulation to improve molecular targeting

**DOI:** 10.1371/journal.pone.0293700

**Published:** 2023-12-20

**Authors:** Francesca Fontana, Alison K. Esser, Christopher Egbulefu, Partha Karmakar, Xinming Su, John S. Allen, Yalin Xu, Jennifer L. Davis, Ariel Gabay, Jingyu Xiang, Kristin A. Kwakwa, Brad Manion, Suzanne Bakewell, Shunqiang Li, Haeseong Park, Gregory M. Lanza, Samuel Achilefu, Katherine N. Weilbaecher

**Affiliations:** 1 Department of Medicine, Washington University School of Medicine, St. Louis, MO, United States of America; 2 Department of Radiology, Washington University School of Medicine, St. Louis, MO, United States of America; 3 Department of Surgery, Washington University School of Medicine, St. Louis, MO, United States of America; Lady Davis Institute for Medical Research, CANADA

## Abstract

**Background:**

Conjugation of transferrin (Tf) to imaging or nanotherapeutic agents is a promising strategy to target breast cancer. Since the efficacy of these biomaterials often depends on the overexpression of the targeted receptor, we set out to survey expression of transferrin receptor (TfR) in primary and metastatic breast cancer samples, including metastases and relapse, and investigate its modulation in experimental models.

**Methods:**

Gene expression was investigated by datamining in twelve publicly-available datasets. Dedicated Tissue microarrays (TMAs) were generated to evaluate matched primary and bone metastases as well as and pre and post chemotherapy tumors from the same patient. TMA were stained with the FDA-approved MRQ-48 antibody against TfR and graded by staining intensity (H-score). Patient-derived xenografts (PDX) and isogenic metastatic mouse models were used to study *in vivo* TfR expression and uptake of transferrin.

**Results:**

*TFRC* gene and protein expression were high in breast cancer of all subtypes and stages, and in 60–85% of bone metastases. TfR was detectable after neoadjuvant chemotherapy, albeit with some variability. Fluorophore-conjugated transferrin iron chelator deferoxamine (DFO) enhanced TfR uptake in human breast cancer cells in vitro and proved transferrin localization at metastatic sites and correlation of tumor burden relative to untreated tumor mice.

**Conclusions:**

TfR is expressed in breast cancer, primary, metastatic, and after neoadjuvant chemotherapy. Variability in expression of TfR suggests that evaluation of the expression of TfR in individual patients could identify the best candidates for targeting. Further, systemic iron chelation with DFO may upregulate receptor expression and improve uptake of therapeutics or tracers that use transferrin as a homing ligand.

## 1. Introduction

In the United states, breast cancer is the second leading cause of cancer death in women [1], in Europe the first [2]. The main cause of death is the development of metastases which cause 5-year survival numbers to drop from 85% [3] to 22–8% [4]. Identification and treatment of systemic disease in breast cancer patients are crucial to improve treatment and survival. Molecular imaging and nanotherapy represent promising strategies to efficiently locate and reach all tumor foci by relying on the over-expression of targetable surface proteins by cancer cells.

Transferrin Receptor (TfR, TfR1, *TFRC*) is a membrane protein commonly overexpressed by cancer cells [5–7], making it an appealing target for anti-cancer biomaterials such as molecular probes or nanoparticles [5, 6, 8–14]. Its physiological function is to allow cellular intake of iron, safely bound to transferrin. While systemic iron metabolism involves high liver expression of transferrin receptor 2 (*TFR2*), TfR is present in all cells, and its expression responds to the cell’s metabolic requirements, for example through transcriptional regulation by iron responsive proteins (IRP1 and IRP2) [15]. To sustain increased iron demand for catalysis and biogenesis [16], tumor cells often overexpress TfR, which can be directly regulated by oncogenic pathways such as c-Myc and Ras [8, 16–18].

Transferrin was identified as a key factor for breast cancer cell survival about 40 years ago [19], and TfR was shown to be upregulated in breast cancer [6, 16] relative to normal breast or fibromas [20, 21]. Since then, preclinical models or early-stage clinical studies have addressed the potential of conjugating transferrin to chemotherapeutics [11, 12], drug-carrying nanoparticles [9, 22–24], and optical [25–27] or radioactive [8, 10] tracers to target breast cancer.

Targeting agents often rely on membrane expression of the receptor in tumor cells, which would bind more ligand than non-tumor cells improving specificity [28]. In order to facilitate translation of transferrin-conjugated probes and therapeutics in breast cancer, we set out to characterize the expression of TfR throughout large datasets and [8, 25, 26] ad-hoc collections of breast cancer histological specimens, with a particular focus on the inclusion of post-chemotherapy and metastatic lesions, which are often underrepresented in discovery studies. Addressing the dynamic relationship between the regulation of the receptor and the uptake of biomaterials, we then asked whether iron-responsive regulation of transferrin could be used to increase the uptake of transferrin conjugates in breast cancer cell lines *in vitro* and improve transferrin accumulation at breast metastatic sites *in vivo*. Our results confirm the frequent overexpression of transferrin receptor in all clinical and pathological classes of breast cancer, but also show variability between patients and even individual cells within the same tumor. Further, in vitro and in vivo evidence suggests that deferoxamine (DFO), an FDA-approved iron chelator under investigation for the treatment of breast cancer [29–33], can enhance uptake of transferrin conjugates by metastatic breast cancer. Overall, this suggests that the efficacy of transferrin receptor targeting may be improved in two ways: (1) by confirming the overexpression in the tumors of individual patients by molecular imaging, and (2) by modulating iron availability to increase expression of TfR by cancer cells.

## 2. Materials and methods

### 2.1 Data mining

Data on cancer vs normal and breast cancer subtypes were obtained from the GENT2 (Gene expression database of Normal and Tumor tissues [34]). Clinical and gene expression data from The Cancer Genome Atlas (TCGA) breast cancer set [35] were downloaded from the UCSC Xena genome explorer [36]. Conversion from gene ID to microarray probes was performed by downloading platform translations from the Gemma database [37]. Datasets GSE153470 [38], GSE114082 [39], GSE21974 [40], GSE18728 [41], GSE111151 [42], GSE58708 [43], GSE62598 [44], GSE130788 [45], GSE165393 [46], GSE175692 [47], and GSE43837 [48] were obtained from the Gene Expression Omnibus (GEO) database [49]. Single cell RNA-sequencing (scRNAseq) breast cancer dataset in Chung et al. [50] was analyzed visualizing clusters based on histology subtype wth Cell Expression Atlas (EMBL-EBI [51]). Data on transferrin receptor 1 (*Tfrc*) and transferrin receptor 2 (*Tfr2*) from normal mouse tissue was obtained for 3 different datasets from the Expression Atlas (www.ebi.ac.uk/gxa) [52].

### 2.2 Tissue banking and consent

Patient samples from the St. Louis Breast Tissue Registry (https://breasttissue.wustl.edu) were obtained and accessed in accordance with the guidelines established by the Washington University’s Institutional Review Board (IRB #201102394) and WAIVER of Elements of Consent as per 45 CFR 46.116 (d). All patient information was deidentified prior to investigator use. All of the human research activities and all activities of the IRBs designated in the Washington University Federal Wide Assurance, regardless of sponsorship, are guided by the ethical principles in "The Belmont Report: Ethical Principles and Guidelines for the Protection of Human Subjects Research of the National Commission for the Protection of Human Subjects of Biomedical and Behavioral Research."

### 2.3 Immunohistochemistry (IHC) and histomorphometry

Tissue microarrays were generated from the St. Louis Breast Tissue Registry (https://breasttissue.wustl.edu) as described in Ross et al. [53] with breast cancer samples and non-breast control tissues, and quality checked by a board-certified pathologist. Samples were hybridized with the MRQ-48 monoclonal anti-human CD71 antibody [54] (FDA-GS1 #00841683101185) produced and validated by eBioscience (cat #14–0718), and developed with the EnVision+ System-HRP (Dako) according to the manufacturer’s protocol. Commercially available tissue microarrays (TMA) #MTU951, #BC081116c, #BC081116c (US BioMax Inc) were used to validate the protocol and showed increased staining in cancer samples relative to matching non-tumor tissue (not shown). Slides were counterstained with hematoxylin, and scanned on the Zeiss AxioScan.Z1 microscope.

Breast cancer subtyping was based on estrogen receptor (ER), progesterone receptor (PR), and human epithelial growth factor receptor 2 (HER2) status, assessed by the diagnosing pathologist, and classifying as luminal A tumors that were ER+,PR±,HER2− (*n* = 39), luminal B for ER+,PR±,HER2+ (*n* = 17), HER2-enriched for ER−,PR−,HER2+ (*n* = 19), and triple-negative (TNBC) for ER−,PR−,HER2− (*n* = 23) tumors. Eight breast carcinomas had incomplete genetic subtyping and were excluded from the subtype analysis.

Tumor-associated TfR (CD71) expression was semi-quantitatively scored using the histo-score (H-score) system: H-score = ∑(i x %) [55], where “i” is the staining intensity (0–3 scale), and “%” is the percentage of tumor cells stained at each intensity.

### 2.4 Animal models

Animal studies were approved by the Washington University Institutional Animal Care and Use Committee (IACUC) and are reported according to the ARRIVE guidelines. TfR expression was assessed in archived tissues from mouse tumor models [53, 56].

For Patient-Derived Xenograft (PDX) experiments, 10 NSG mice were implanted with WHIM68 and 9 with WHIM69 breast cancer cell lines subcutaneously as previously described [57] and treated with Taxol (30mg/kg) and carboplatin (50mg/kg) or vehicle. Mice implanted with WHIM68 were treated on days 30, 37, and 44; mice implanted with WHIM69 on days 44, 51, and 58. Eight mice implanted with WHIM68 (N = 5 vehicle and N = 3 NAC) were sacrificed on day 57, and mice with regrowing tumors (relapse N = 2) were euthanized on day 148. Mice injected with WHIM69 (N = 5) and treated with vehihcle (N = 3) were sacrificed on day 62, while NAC mice were sacrificed on day 111 (N = 2), and relapse at day 192 (N = 2) [57].

To establish diffuse/metastatic triple negative breast cancer 0.5x10^5^ 4T1 tumor cells were injected during general anesthesia into the left ventricle 6-week-old BALB/cJ (4T1) female mice (Jackson Laboratories). For diffuse luminal B cancer, 0.5x10^5^ MMTV-Bo1-Luc-GFP cells were injected in the left ventricle of C57Bl/6 female mice of 5–6 weeks of age (under general anesthesia). Development of metastases was monitored in vivo total body BLI.

Ferritin was measured in serum samples by ELISA according to manufacturer protocol (Mouse Ferritin ELISA kit, Crystal Chem, Elk Grove Village, IL), and optical density was recorded with a SpectraMax microplate reader (Molecular Devices, San Jose, CA).

### 2.5 In vivo and ex-vivo imaging

Transferrin-Vivo 750 (PerkinElmer, Waltham, MA, referred to as AF750-Tf) was injected i.v. according to manufacturer instructions 24 hours prior to sacrifice and dual BLI/optical imaging. Average radiance (photons/sec/cm^2^) and average radiant efficiency were measured from fixed regions of interest (ROIs) using Living Image 2.6 (PerkinElmer, Waltham, MA).

Ex-vivo optical imaging of organs and tissues was performed and average radiant efficiency was measured in the heart, lungs, spleen, kidneys, liver, gut, brain, white adipose tissue (WAT) deposits in the abdomen (gonadal) and subcutaneous (including the mammary fat pad), brown adipose tissue (BAT) from the intrascapular region, muscles (femoral quadriceps), and bones (legs, spine, pelvic gridle).

At 11–12 days post implantation, 4T1 tumor-bearing and non-tumor bearing mice (N = 5) were injected i.v. with Transferrin-AlexaFluor 680 (ThermoFisher, Waltham, MA) imaging agent and then followed by mice imaging at 0, 2, 6 and 24 h time-points using the fluorescence molecular tomography (FMT 4000, PerkinElmer, Waltham, MA) and the Pearl NIR small animal imager (Li-Cor Biosciences Inc., Lincoln, NE, USA) in vivo. Following euthanasia with 5% isoflurane (as in American Medical Veterinary Association guidelines) ex-vivo imaging was performed at 6h or 24h using the Pearl NIR imager and the IVIS50 bioluminescence image (PerkinElmer, Waltham, MA) as previously described [58]. The mean fluorescence intensity (MFI) and the total photon flux (photons/sec) for each organ was estimated using the Pearl and IVIS50 software, respectively and summarized using a bar plot ± SEM (n = 5 per group).

Deferoxamine mesylate (Sigma, DFO) was administered by i.p. injection. Lack of treatment-related toxicity was verified in two preliminary experiments by administering to C57Bl/6 100mg/kg (N = 2) versus 200mg/kg daily (N = 2) for 8 days.

To model the effects of DFO treatment in. Mice were randomized to receive vehicle (HBSS, N = 4-5/experiment, total N = 9) or DFO 200mg/kg (N = 5-6/experiment, total N = 11).

### 2.6 In vitro experiments

The murine C57BL/6 PyMT-Bo1 luminal B breast cancer and 4T1-GFP-Luc cell line expressing green fluorescent protein (GFP) and firefly luciferase (Luc) were originated and tested as described (Su [58] and David Piwnica-Worms lab [59]). T4D1 and MCF-7 were obtained from ATCC. Low-passage stocks were used and regularly tested for Mycoplasma and maintenance of growth characteristics. Modulation of extracellular iron was obtained in vitro by treating cells with Ferric Ammonium Citrate (FAC, Sigma) or Deferoxamine (DFO, Sigma) 80-200μmol/L for 24 to 48h. Transferrin uptake in vitro was assessed by incubation with transferrin from human serum Alexa Fluor^TM^ 488 conjugate (Thermo Fisher T13342) according to manufacturer’s instructions and analyzed for fluorescence excitation/emission wavelengths of 485/506 nm with a SpectraMax microplate reader (Molecular Devices, San Jose, CA). For normalization, nuclei were counter-stained with Hoechst (co-registering fluorescence at 361-450nm) or by crystal violet measuring 570nm optical density. PhRodo^TM^ Red transferrin conjugate (Thermo Fisher, P35376) and nuclear staining with Hoechst were used for assessment of endocytosis of Tf. Live cell images were acquired with an inverted stage microscope (Nikon E800, DIAPHOT300), and images merged with ImageJ (imagej.nih.gov/ij).

### 2.7 Statistics

Power analysis was performed using G*power 3 [35] for sample size determination of two group comparisons [60]. For power of multi-group analyses, we used the open-access sample size calculator v1.058 (https://homepage.univie.ac.at/robin.ristl/samplesize.php). For the tumor microarray, a priori analysis for allocation ratio of 9 tumor/non tumor indicated that a sample size of 94 or higher would detect a doubling of the H-score with 80% power and α = 0.05. For in vivo experiments, differences of 50% with 25% standard deviations in continuous endpoints of two independent groups of 0.7–1 ratio would be detected for 4–6 mice per group at 80–90% power with α = 0.05.

Pearson’s correlation and linear regression were used for normally distributed data. Differences among experimental groups were analyzed using a two-tailed t-test, Fisher’s exact test, one-way or two-way ANOVA (with post-hoc tests). For non-normally distributed data, differences among experimental groups were analyzed using a two-tailed Mann–Whitney U-test for unpaired samples, and correlations tested by Spearman’s test. All tests were considered significant at P≤0.05. Unless otherwise specified, gene expression data are shown as boxplots with mean and range, and bars as mean and standard deviation (mean ± SD. Prism 9 (GraphPad Software) was used for data analysis and graph preparation.

## 3. Results

### 3.1 Expression of TfR1 in primary breast cancer

The transferrin receptor (TfR) is necessary for the survival of most cells; however, several reports have indicated that it is often substantially overexpressed in cancer [19–21]. To investigate clinical and pathological association of TfR expression in breast cancer, we used data mining and immunohistochemistry on tissue microarrays (TMA).

Data on gene expression were obtained from the Gene Expression database for both normal and tumor tissue [34] (GENT2) combining GEO-deposited data from Affymetrix U133A or U133Plus2 arrays, and data from the cancer genome atlas (TCGA), which combines sequencing and microarray data from several platforms.

Both the GENT2 and TCGA breast cancer data sets showed increased gene expression of *TFRC* (p<0.0001) in breast cancer (N = 5574 and N = 1210) relative to normal breast (N = 475 and N = 113 respectively, S1A and S1B Fig).

To investigate associations with tumor staging, the samples from the TCGA dataset were stratified according to the parameters of the American Joint Committee on Cancer (AJCC) TNM (tumor, nodes, metastasis) system. No differences were found in the transcript levels of *TFRC* between T1, T2, T3, or T4 patients (P = 0.1), or according to N score (P = 0.4, ANOVA). Only 2.4% of patients in the TCGA dataset were metastatic at diagnosis, but no trend for differences in *TFRC* expression between M0 or M1 was noted (t-test, P>0.9). Accordingly, no differences were seen in *TFRC* RNA across stages I to IV (ANOVA, α = 0.05, 1-β = 0.21) (S1 Table).

The GENT2 dataset was stratified according to histological subtype, annotated for 1467 patients. Expression of *TFRC* was found in all subtypes, but with significant quantitative differences across types, with Luminal A type having lowest levels and TNBC the highest (P<0.001, α = 0.001, 1-β≅1). Stratification of the GENT2 dataset by grade also showed increase in *TFRC* gene expression across pathological grades (P<0.0001, 1-β≅1) [21].

Multivariate analysis of 1097 tumor samples in the TCGA dataset showed a significant correlation between *TFRC* and markers of proliferation, such as *MKI67* (Ki67), *CCNB1*, *CCNA2*, *CDK2*, *CDK6*, *TOP2A*, and *TOP1* (P<1E-20, r>0.3). Also, *TFRC* was negatively correlated with estrogen receptor *ESR1*. *TFRC* correlation was relatively weak with *MYC* (P = 0.003, r = 0.09), but highly significant with *NRAS* (P = 3.8E-21, r = 0.28) and *KRAS* (P = 3.2E-27, r = 0.32), recently associated with aggressive TNBC [61]. These results are in line with previous reports on *TFRC* expression being positively correlated with proliferation [62] and negatively with ER [61, 63].

**Fig 1:**
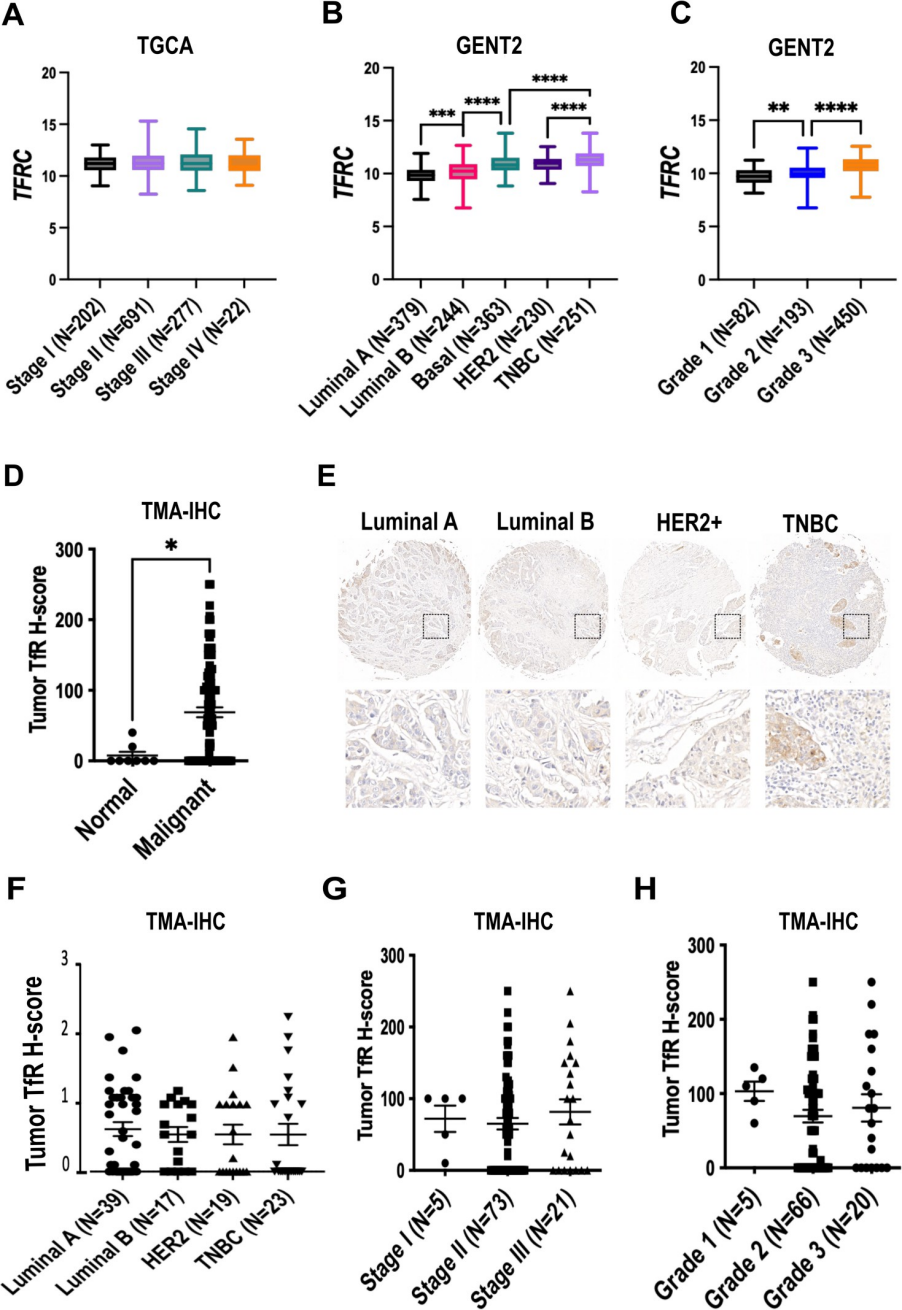
Expression of TfR in primary breast tumors. A-C) Gene expression of *TFRC* A) by stage (TGCA, total N = 1192), B) by histological subtype (GENT2, total N = 1467), C) by grade (GENT2, total N = 725). D-H) TfR immunohistochemistry (IHC) D) H-score of normal breast (outside of tumor margin, N = 8) versus malignant breast tumors (N = 99), E) representative images of breast cancer histological subtypes, F) H-score by subtype (total N = 98), G) H-score by stage (N = 99), H) H-score by grade (N = 91). *P<0.05 T-test, ***P<0.001, ****P<0.0001 one-way ANOVA-Tukey’s post-hoc test.

Protein expression of TfR in normal and malignant breast tissue was assessed by immunohistochemistry using a tissue microarray (TMA) of 107 human samples (99 breast cancer and 8 adjacent normal breast), stained for TfR with the FDA-approved anti-CD71 antibody MRQ-48, and scored taking as reference the darkest stain (highest expression) present within each tissue sample (S1D Fig). Unlike the continuum offered by transcriptomic analysis, IHC allowed to identify expression of TfR at significantly higher levels than background in 62/99 (62.6%) of breast cancer versus 2/8 (25%) of control breast tissue. This is consistent with previous reports showing by IHC expression in 70% of breast cancer biopsies [20, 63]. Semi-quantitative assessment showed that TfR expression was significantly increased in breast cancer compared to non-malignant adjacent breast tissue (average H-score 70±7; 8±5, P<0.05) (Fig 1D).

Next, breast cancer samples were stratified according to histological subtype, grade, and staging. TfR staining was detectable in 25/39 (64%) Luminal A, 12/17 (71%) Luminal B,10/19 (53%) HER2, and 10/23 (43%) TNBC. IHC intensity score showed high intra-group variability, but no noticeable trend across subtypes of breast cancer (P = 0.75, 1-β = 0.07).

Next, TMA biopsies were stratified according to the TNM system. TfR staining was observed in 5/6 (83%) T1 tumors, 41/66 (62%) T2, 5/9 (53%) T3, and 11/16 (69%) T4. Staining intensity was variable, but showed no significant inter-group differences (P = 0.5, 1-β = 0.8) (S1E Fig). The tumor H-score appeared higher in the 5 patients classified as N2 (138±43) relative to N0 (71±8), or N1 (52±15), likely due to unequal group composition and low statistical power (1-β = 0.54) (S1F Fig). Classification by stage showed consistent TfR expression, with no apparent difference in staining intensity between stage I (N = 5, H score 72±41), stage II (N = 73, 65±68), and stage III (N = 21, 81±80, P = 0.56, 1-β = 0.06, Fig 1G).

Analysis of the TMA by tumor grade showed TfR staining in all groups (Fig 1H), including grade 1 (N = 5, H score 103±29), grade 2 (N = 66, 70±80), and grade 3 (N = 20, 81±82), with no detectable differences in staining intensity (P = 0.38, 1-β = 0.13).

Overall, transcriptomic data, more quantitative and available for large number of cases, allowed to confirm previous reports of higher expression in more aggressive grades [21, 64, 65] and in TNBC [21, 63–65] (Fig 1B and 1C), and correlations with molecular markers of proliferation [21, 64–66] (S1C Fig). Immunohistochemistry showed high prevalence of TfR staining, with heterogeneity across tumors and even within the same tumor. Notably, high TfR protein expression could occur even in subtypes generally associated with lower transcript levels, such as ER+ or low-grade tumors. This suggests that the selection of cases eligible for TfR-targeted therapeutics should be based on the expression of TfR in the specific tumor, rather than on the histological subtype or grade.

### 3.2 Breast cancer expression of transferrin receptor after chemotherapy

Exposure to chemotherapy can induce profound changes in tumor cell metabolism and regulation of membrane receptors. To investigate the effects of pharmacological treatment on the expression of TfR, we used datamining, built a dedicated tumor microarray, and performed IHC on chemotherapy-treated patient-derived xenografts (PDX).

In the TCGA dataset, we found 53 samples annotated as having received additional chemotherapy, versus the other 1044 primary breast tumors. *TFRC* expression did not show differences between the two groups (P = 0.15), but unequal group composition and heterogeneity of therapeutic regimens may limit the statistical power and biological significance (1-β = 0.19) (S2A Fig).

**Fig 2:**
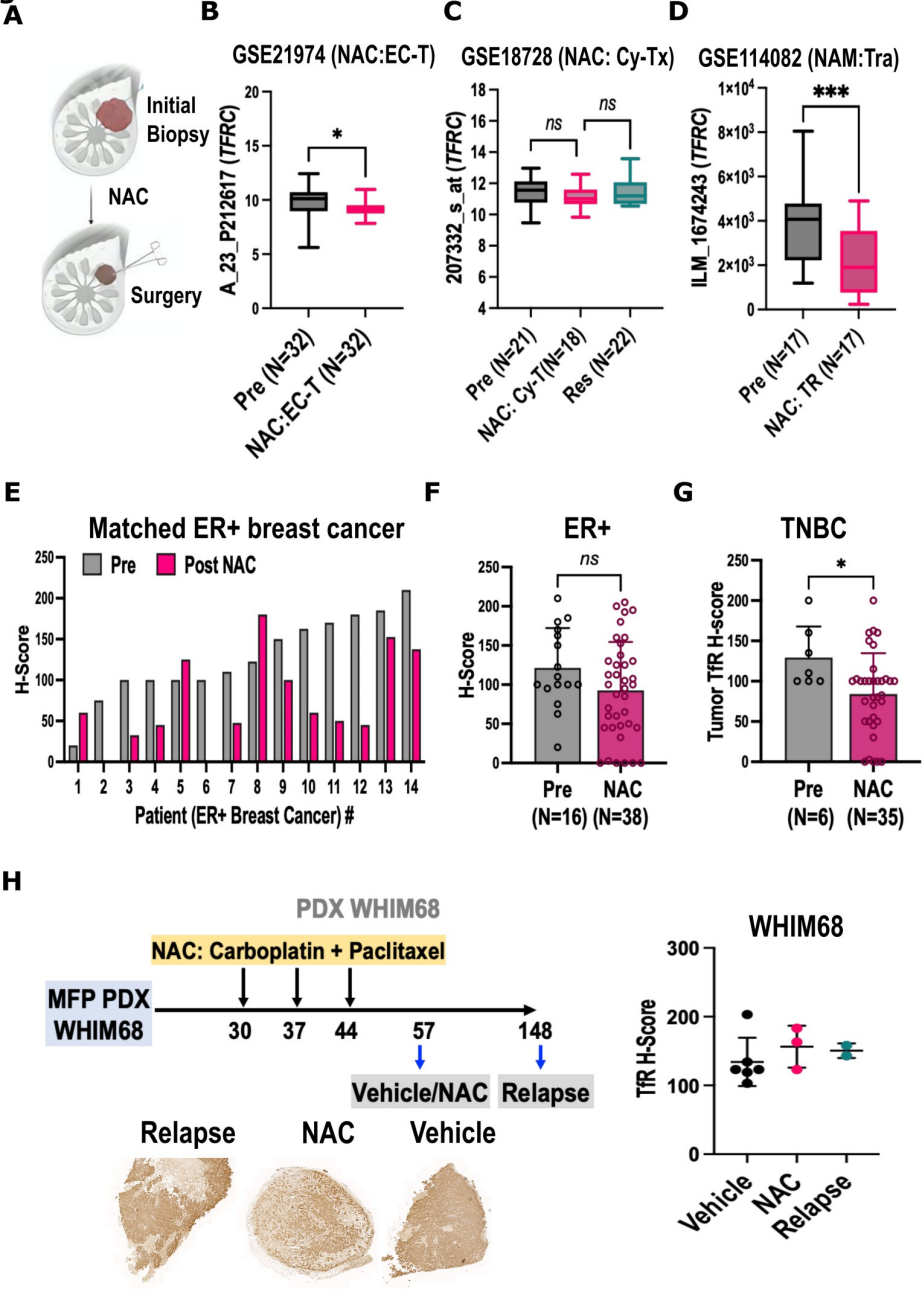
Expression of TfR after neoadjuvant therapy. A) experimental design: unless otherwise specified pre- refers to tissue from the diagnostic biopsy, post- refers to tissue from the resected tumor; B) *TFRC* expression in GSE21974: 32 patients sampled before and after four cycles of epirubicin and cyclophosphamide prior to taxane (EC-T), C) *TFRC* expression in GSE18728 (N = 22 patients): biopsy samples collected at diagnosis (Pre) and after one cycle of capecitabine and docetaxel (NAC: Cy-TX), and from the resected tumor (Res); D) *TFRC* expression in GSE114082 (N = 17 patients) pre- and after trastuzumab (NAC:TR). E-G) Tumor microarray IHC TfR score E) waterfall plot of patient-matched ER+ breast cancer (BC) before and after NAC (N-14), F-G) H-score in (non-matched) samples from diagnostic punch biopsy (Pre, grey), resected tumors (Post-NAC, red), and regional lymph nodes (R-LN, teal) from F) ER+ patients (N = 38) and G) TNBC patients (N = 35. H) TfR IHC of PDX WHIM68, treated with paclitaxel (30mg/kg) and carboplatin (50mg/kg) (Carbotaxol) versus vehicle, and harvested after treatment and at relapse; treatment scheme, representative images of TfR staining and quantification scatter plot post-vehicle, post-NAC, and at relapse. *P<0.05 by paired T-test, **P<0.01 by ANOVA and Tukey’s post-hoc test.

We therefore searched Gene Exprexsion Omnibus (GEO) for transcriptomic data from breast cancer biopsies collected at diagnosis versus after treatment with neoadjuvant chemotherapy (NAC) or endocrine therapy (NAE) (Fig 2A). Positive controls for response to treatment were selected based on the literature. Effects of anthracyclines [67] or taxanes [68] would upregulate p21 (*CDK1NA*); aromatase inhibitors [69] and trastuzumab [70] would downregulate *CCDN1*.

In the GSE21974 dataset [40], profiling naïve breast tumors versus treated with four cycles of epirubicin and cyclophosphamide, *CDK1NA* was increased in treated patients (P<0.01, S2B Fig). However, *TFRC* expression showed no difference (Fig 2B). The GSE18728 dataset [41] includes patients treated with capecitabine and docetaxel, sampled before (N = 21), after 1 cycle of NAC (N = 18), and at resection (N = 22). In this set, no significant changes were noted in *TFRC* expression between treated and untreated tumors (Fig 2C), unlike *CDK1NA* (P<0.05 S2C Fig). In the GSE28826 series [71], matched tumor samples were obtained before (N = 14) and after (N = 14) NAC with taxanes and anthracyclines. Again, sample size was adequate to show *CDKN1A* increase, but no differences were found in *TFRC* transcription (S2D and S2E Fig).

Effects of neoadjuvant endocrine therapy (NAE) were assessed on the dataset GSE153470 [38], profiling tumors from 81 post-menopausal women before and after treatment with aromatase inhibitors (AI). *CCND1* was significantly downregulated after AI (P<0.001, S2F Fig), but *TFRC* expression showed no difference between pre- and post-NAE samples (S2G Fig).

In GSE114082 [39], profiling 17 HER2+ tumors before and after treatment with trastuzumab, CCND1 was decreased as predicted for antibody inhibition of HER2 [70] (S2H Fig). Unlike other treatments, however, trastuzumab treated tumors also had a significant decrease in *TFRC* expression relative to baseline (Fig 2D). The dataset GSE130788, relative to the TRIO-B-07 clinical trial (NCT#00769470) [45], compared the gene expression profiles of HER2+ breast tumors (N = 199 samples) at baseline and after three trastuzumab-based NAC regimens (S2I Fig). In this more comprehensive multitherapy study, tumors sampled after treatment had significantly lower expression of *TFRC* (Two-way ANOVA, treatment P<0.0001, 1-β≅1). Decrease of expression of *TFRC* upon treatment with trastuzumab (alone or with chemotherapy) is consistent with the role of EGFR signaling in regulating iron homeostasis [17].

Overall, data mining shows that, with the exception of HER2 targeting, neoadjuvant chemotherapy does not negatively impact the expression of *TFRC*.

Custom-made tissue microarrays (TMA) were prepared from 72 de-identified biopsies from ER+ and 56 from TNBC patients (all comers, all treatment protocols), and stained for IHC of TfR.

For ER+ tumors, matching Pre- and Post-NAC (all protocols) biopsies were available and for 14 patients (Fig 2E), for TNBC 6 (S2I Fig). Comparison of matched pre- and post-NAC tumors showed a significant reduction in H-Score of TfR staining in ER+ (p<0.005, Fig 2E), and an average 30% reduction in TNBC (S2I Fig) that did not reach significance (N = 6, P = 0.09, 1-β = 0.33). However, 12/14 ER+ (85%, Fig 2E) and 6/6 TNBC (100%) were still positive for TfR staining.

The complete sets of ER+ (Fig 2F) and TNBC (Fig 2G) perioperative samples, not matched by patient, comprised naïve tumors (N = 16 ER+ and N = 6 TNBC), NAC-treated tumors (N = 38 ER+ and N = 35 TNBC), and regional lymph nodes (N = 19 in ER+ and N = 15 TNBC). Naïve tumors showed the highest H-scores in both ER+ (121±51) and TNBC (134±40) TMA. In comparison, lymph nodes showed significantly lower TfR staining, both in ER+(P = 004, 1-β = 0.77) and TNBC (P = 0.002, 1-β = 0.87). In the ER+ TMA, 16/16 (100%) of naïve, 35/38 (87%), and 15/18 regional lymph nodes (83%) showed above-threshold TfR staining.

Patient derived xenograft (PDX) models offer the opportunity to study human tumors in vivo in highly controlled conditions. This allows the flexibility of an experimental model, for example matched vehicle/drug controls or the study of untreated relapse, while maintaining impressive similarity in drug sensitivity relative to the patients donating the tissue [72].

Here, we studied TfR expression in NSG mice inoculated with two TNBC PDX lines, WHIM68 (Fig 2H) and WHIM69 (S3 Fig), derived from the same patient respectively before and after NAC with docetaxel and carboplatin [72]. Mice with engrafted PDX tumors were treated with three cycles of carboplatin and paclitaxel (NAC) versus vehicle (Vh), and euthanized to collect tumors after treatment or upon relapse. Similar to previous reports [8], TfR staining was detectable in all PDX tumors at baseline. Moreover, both in WHIM68 and WHIM69 tumors, TfR expression was maintained after NAC, or at distant relapse, with low variability and high H-score (Figs 2H and S3).

Overall, this data suggest that, albeit at variable levels, TfR is expressed in the majority of the breast cancer cells that remain after neoadjuvant chemotherapy.

### 3.3 Expression of transferrin receptor in human breast cancer metastases

Breast cancer metastases are responsible for the majority of breast cancer mortality and affect several organs, making their detection and systemic treatment a priority for biotechnological research. Bones are affected in 75% of metastatic cases (as only metastatic site in 40%), followed by lungs, lymph nodes, liver, and brain [4]. In order to evaluate expression of TfR by human breast cancer metastases, multi-organ and brain metastases datasets were analyzed, and a tissue microarray of matched bone metastases and primary breast tumors was generated.

**Fig 3:**
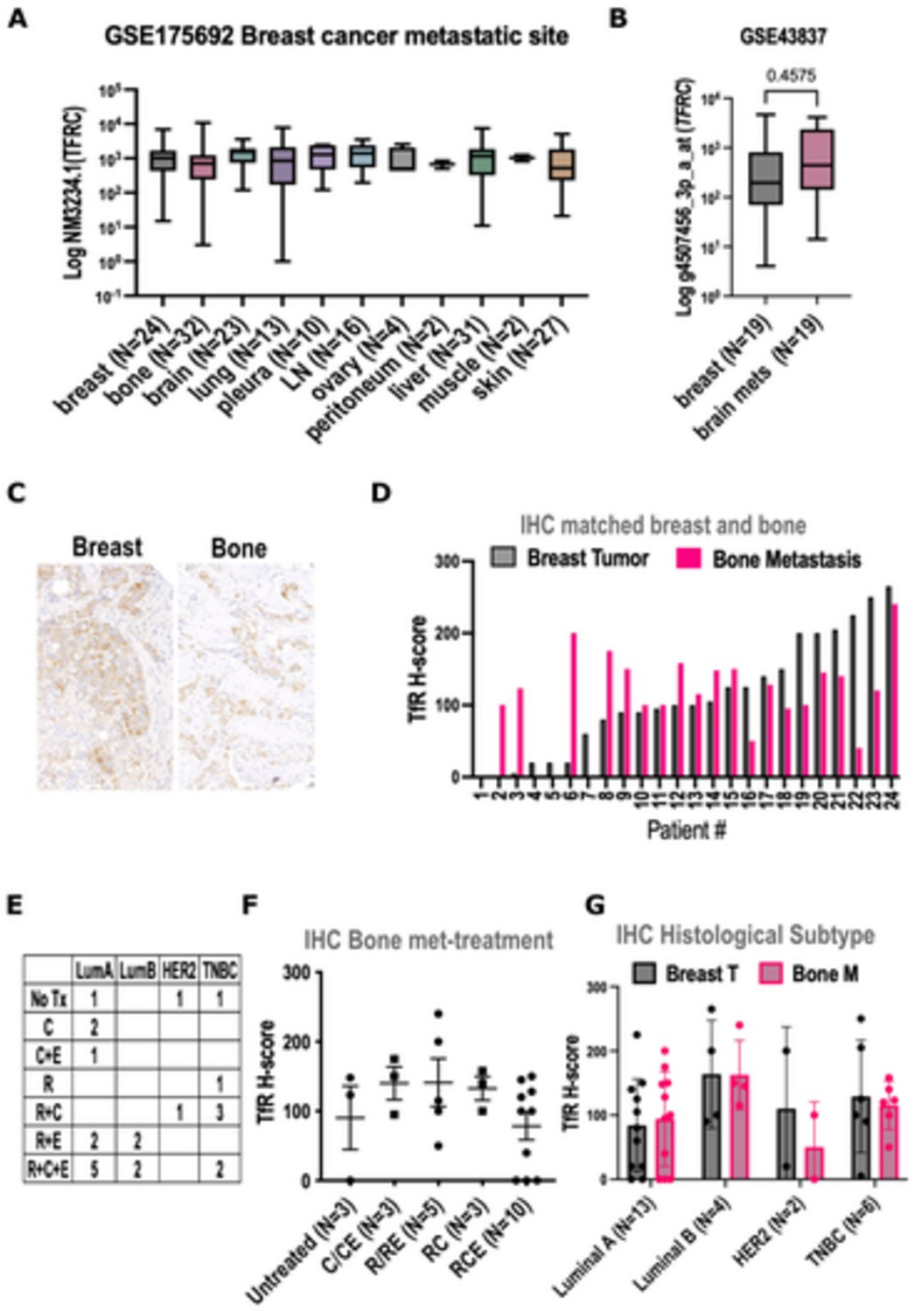
TfR expression in breast cancer metastases. A) *TFRC* expression in GSE175692 biopsies from different metastatic sites (total N = 117), B) *TFRC* expression in GSE43837 of matched primary breast cancer and brain metastases samples (N = 18 patients). C-G) TMA TfR staining of primary breast cancer and bone metastases C) representative image, D) waterfall plot of 28 patient-matched primary tumor and bone metastasis samples (black breast, red bone); E-G) previous treatments at the time of metastasis in TMA samples: No Tx = no treatment, C = chemotherapy, C+E = chemotherapy and endocrine therapy, R = radiation therapy, R+C = radiation and chemotherapy, R+E = radiation and endocrine therapy, R+C+E = radio- chemio- and endocrine therapy, E) tumor subtypes vs treatments, LumA = luminal A, LumB = luminal B, HER2 = Her2-high, TNBC = triple negative breast cancer, cells: number, F) H-Score of bone metastases by treatment (tot N = 24). G) H-Score of bone metastases by histological subtype (tot N = 25).

*TFRC* expression was analyzed in the GSE175692 dataset [47], comprising 184 samples of human breast cancer metastases, which showed consistent expression and no difference across 11 metastatic sites (Fig 3A). Similarly, in the GSE43837 dataset, *TFRC* was expressed at comparable levels between primary breast cancer and brain metastases (Fig 3B) [48].

A dedicated TMA was prepared with matched primary breast cancers, sampled at diagnosis, and bone metastases from 24 patients (Fig 3C–3E). Again, we found that 22/24 (92%) of primary breast cancers in this array expressed TfR. Also 20/24 (83%) of bone metastases expressed TfR (Fig 3C and 3D), with no correlation of the H-score relative to the original tumor (plot not shown). As expected, 21/24 patients had received systemic treatment prior to the appearance of metastases, but no association was found between type of treatment and TfR H-Score (Fig 3E). Staining for TfR was positive in 9/11 (81%) primary tumors and 8/11 (73%) bone metastases from Luminal A breast cancer. Both primary HER2+ tumors (2/2) were positive for TfR, but only the untreated patient showed TfR staining at the metastatic site. TfR staining was positive in both primary and bone biopsies from 4/4 Luminal B and 6/6 TNBC patients. Semi-quantitative assessment of staining intensity showed no significant inter-group differences (ANOVA 1-β = 0.28).

These findings confirm that, similar to primary tumors, most breast cancer metastases express high levels of transferrin receptors, though at variable levels.

### 3.4 Expression of transferrin receptor in mouse models of breast cancer metastases

In order to establish whether syngeneic mouse models of metastatic breast cancer replicate the variable pattern of expression of TfR, murine breast cancer models were investigated by datamining and histology.

In GSE165393, RNAseq identified *tfrc* transcripts in all MMTV-PyMT tumor sites (S3B Fig), and GSE62598 [73] in all 4T1 tumors or metastases (S3C Fig). Neither dataset had statistical power to test inter-group differences (1-β = 0.2 and 1-β = 0.3), but both showed variability in expression within and between groups.

To evaluate TfR by IHC, a TMA was prepared with samples of mammary fat pad (MFP) tumors and metastases from mice inoculated with the Bo1 luminal B breast cancer cell line [58] (S3D Fig), or the 4T1 TNBC (S3E and S3F Fig). TfR expression was detectable in all cancer tissues, particularly in Bo1 metastases (S3D Fig), with large variability between samples and groups.

Overall, mouse models of breast cancer mirrored the prevalence and variability of TfR expression at metastatic sites that is observed in humans.

### 3.5 Uptake of transferrin in murine model of triple negative breast cancer metastases

Previous studies on the use of transferrin conjugates in breast cancer considered primary breast tumors [8, 24]. Here, we set out to study the distribution of transferrin conjugates to breast cancer metastases, utilizing commercially available fluorescent transferrin consisting of stable conjugates of recombinant transferrin to AlexaFluor-750 (AF750-Tf) or AlexaFluor-680 (AF680-Tf).

**Fig 4:**
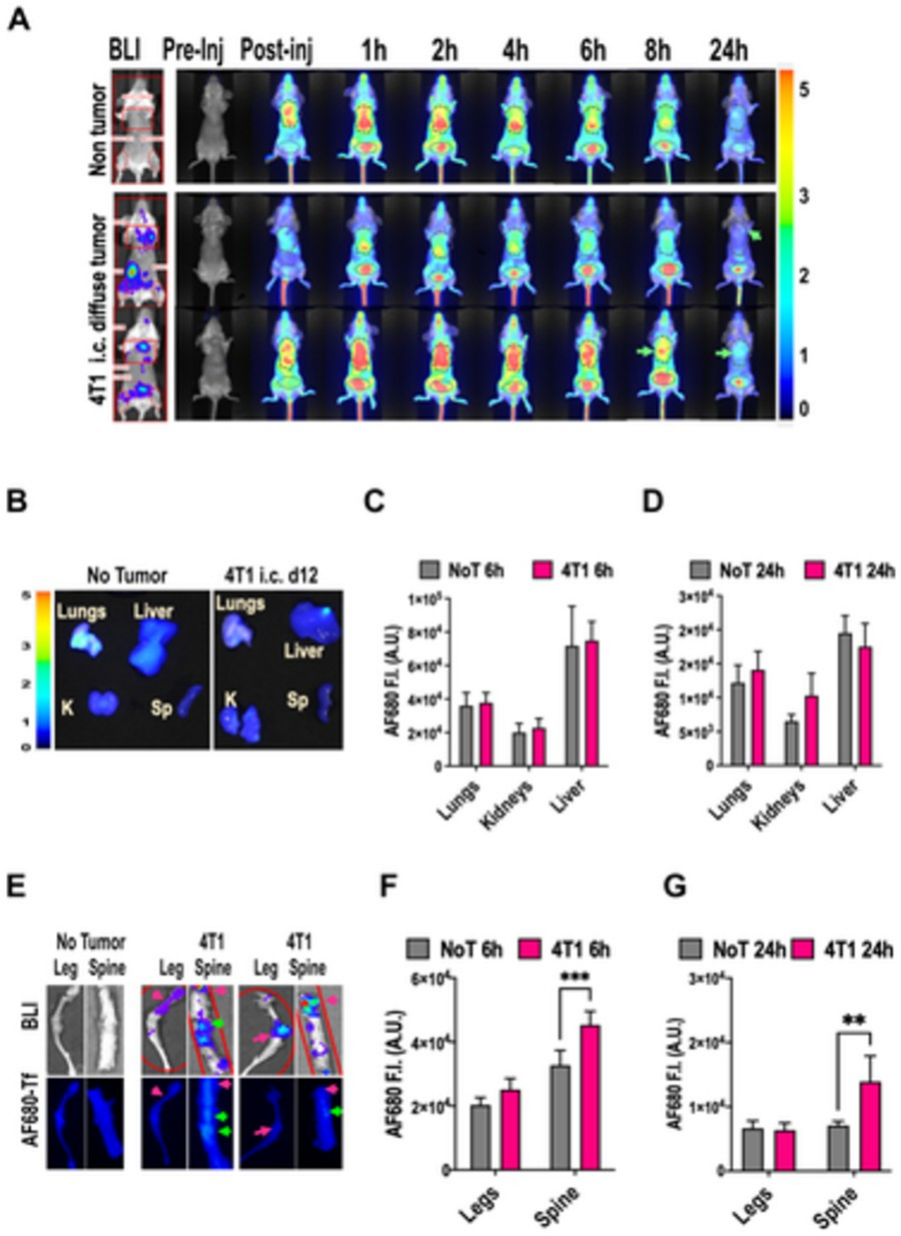
Biodistribution of AF680-Tf in 4T1 metastatic model versus non-tumor Balb/c mice. A) In vivo imaging over time relative to injection of AF680 in one non-tumor (top) and two tumor bearing mice (bottom), left: BLI at t = 24h tumor sites, right: PerL imaging, B) ex-vivo PerL imaging for AF680 of lungs, kidneys (K), liver, and spleen (Sp) of normal (left) versus 4T1 tumor-bearing mice (right); C-D) AF680-Tf fluorescence intensity (F.I.) in arbitrary units (A.U.) of group 1 organs of non tumor (grey, NoT) versus tumor (red, 4T1) mice at C) 6h from injection (N = 4 non tumor and N = 5 tumor) and D) 24h from injection (N = 3 non tumor, N = 4 tumor); E) representative ex-vivo BLI (top) and AT680-Tf imaging (bottom) of hindlimb bones (Leg) and spine of one non-tumor (left) and two 4T1 tumor bearing mice (right); F-G) AF680-Tf fluorescence intensity (F.I.) in arbitrary units (A.U.) of bones of non tumor (grey, NoT) versus tumor (red, 4T1) mice at F) 6h from injection (N = 4 non tumor and N = 5 tumor) and G) 24h from injection (N = 3 non tumor, N = 4 tumor). **P<0.01 by two-way ANOVA and Šidák test.

In order to establish the background accumulation of AF750-Tf and its relationship with expression of its receptors, we compared imaging of non-tumor mice and data on gene expression. Female C57Bl/6 mice were injected with transferrin-750 (AF750-Tf) or vehicle (PBS), and underwent serial in vivo optical imaging (IVIS, Perkin Elmer). In AF750-Tf injected mice,at 6 and 24h hours high fluorescence was recorded the regions corresponding to the liverand in the urinary bladder in vivo (S4A Fig). Ex-vivo imaging showed accumulation of AF750-Tf primarily in the liver, though significant fluorescence was also detected in several organs, such as lungs and kidneys (S4A Fig). Baseline tissue expression of *tfrc* and *tfr2* in mice was obtained from three datasets from the Gene Expression Atlas (https://www.ebi.ac.uk/gxa, S4C-S4E Fig). The average sum of *Tfrc* and *Tfr2* TPM for each organ was then compared to the average radiant efficiency measured ex vivo after injection of AF750-Tf. Despite the use of multiple and independent data sets for both variables, we found a strong positive correlation (p = 0.0005, r = 0.85) between uptake of AF750-Tf and gene expression of its receptors in the same tissue (Fig 4A). Hence, in our system physiologically high expression of TfR and TfR2 can determine high uptake of Tf-conjugates at the organ level, translating into high background for the detection of tumor lesions.

The metastatic triple negative breast cancer model 4T1 was then compared to healthy BALB/cj controls. Uptake of persistent luminescence (PerL) conjugates of transferrin was evaluated 12 days after cardiac injection, in vivo (Fig 4A) and ex vivo (Fig 4B–4F) [74]. Systemic tumor engraftment was verified by total-body BLI (Figs 4A and S4F). In both 4T1 tumor and non tumor-bearing mice, serial in vivo 700nm LiCor Perl scans showed high fluorescence in the area of the liver and bladder (Fig 4A), consistent with previous results (S4A Fig) and published work [75]. Based pharmacokinetics (not shown), one cohort of mice was sacrificed 6h after AF680-Tf injection, and one after 24h. Ex. vivo optical imaging showed broad distribution of labeled transferrin, with high levels in lungs, liver, and kidneys, for both non-tumor and tumor mice. At these sites, fluorescence intensity in 4T1 and non tumor-bearing mice was no higher than in control organs (Fig 4B–4D) in either the 6h (Fig 4C) or 24h (Fig 4D) groups, even when BLI showed significant tumor burden (S4G-S4H Fig). Uptake of AF680-Tf was significantly increased in the vertebrae of tumor bearing mice (Fig 4E–4G), both in the 6h (P<0.001) and 24h (P<0.01) groups, consistent with bone metastases (Fig 4E) detected by BLI. In the leg bones, where BLI showed a more variable and less discrete pattern of metastatic growth, fluorescence intensity was non-superior to non-tumor controls.

Overall, we found that at metastatic sites the uptake of labeled transferrin may be variable, and that high physiological expression of TfR and TfR2 by target organs can affect the ability of Tf-conjugates to identify tumor sites, particularly for smaller lesions.

### 3.6 Extracellular iron regulates uptake of transferrin by breast cancer cells in vitro

Molecular targeting depends on the availability of homing receptors. In the 3’UTR of *TFRC*, an iron responsive element (IRE) allows fine control of its expression. Previous work showed that breast cancer cells downregulate TfR when iron is overabundant, and overexpress it upon iron chelation [76, 77]. We therefore asked whether this mechanism translates to uptake of transferrin conjugates, and whether treatment with FDA-approved iron chelator deferoxamine (DFO) could be used to improve targeting through TfR.

**Fig 5:**
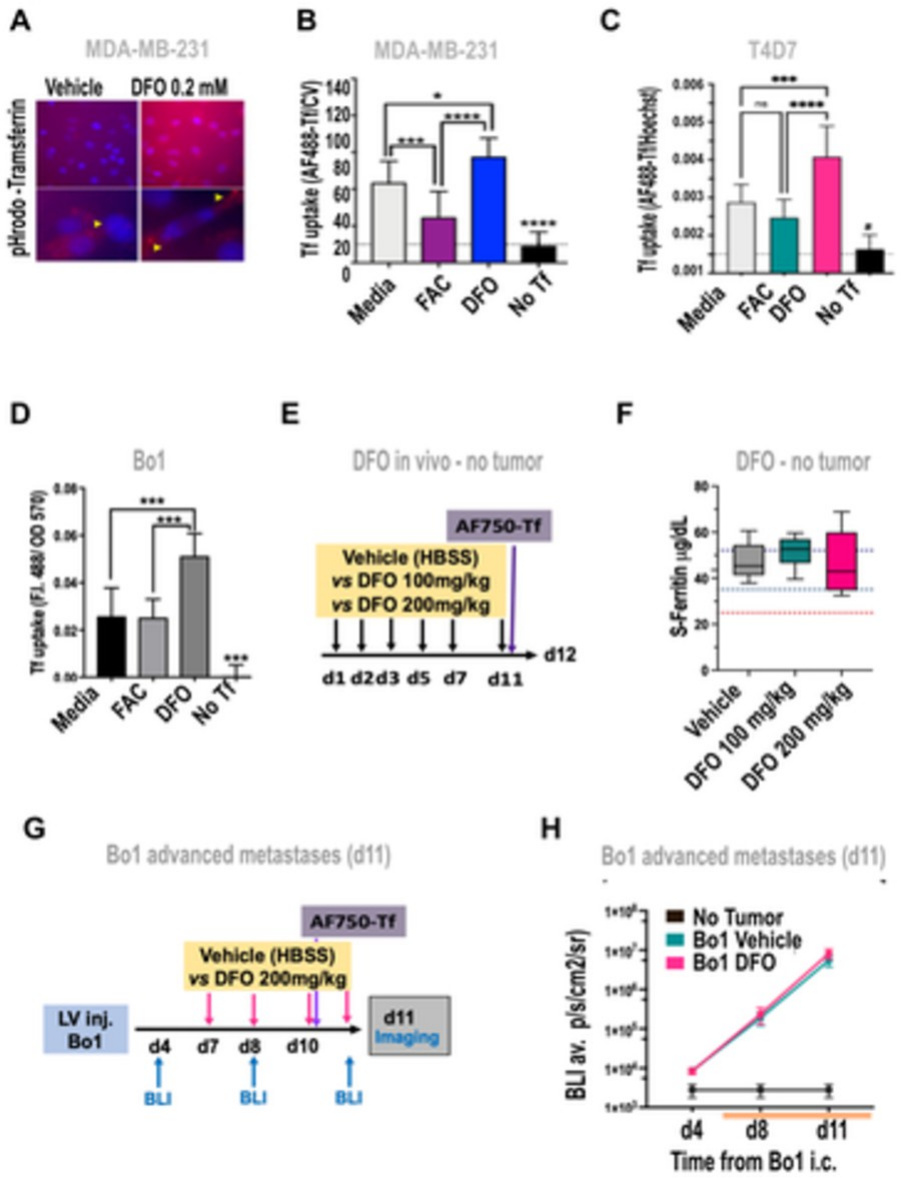
Non-toxic iron chelation upregulates transferrin uptake in breast cancer cells *in vitro* but does not affect tumor growth *in vivo*. A-D) *In vitro* uptake of transferrin conjugates in breast cancer cells A) live imaging of Phrodo Red transferrin conjugate uptake MDA-MB-231 treated with vehicle (left) or DFO 200μM for 48h (right); B) uptake of Tf by fluorimetry of MDA-MB-231 cells pre-treated with FAC 200μM, or DFO 200μM for 48h, ex485/em515nm fluorescence divided by 570 nm optical density after nuclear staining with crystal violet (CV), C) T47D cells pre-treated with FAC 200μM, or DFO 200μM for 24h, ratio of fluorescence intensity at ex485/em515 nm (AF488-Tf) and Hoechst (ex350/em461 nm). D) AF488-Tf uptake by Bo1 cells treated with 100 μM DFO or FAC vs vehicle (AF488-Tf/CV as above); No Tf: background control of cells not exposed to fluorescent transferrin; *P<0.05, ***P<0.001, ****P<0.0001, ns non significant; one-way ANOVA and Tukey’s post-hoc test. E-F) Effect of treatment with DFO in vivo on serum ferritin in mice, E) treatment diagram: non-tumor bearing mice (N = 5) were injected i.m. six times over 11 days with vehicle (HBSS), DFO 100mg/kg, or DFO 200mg/kg, and i.v. AF750-Tf on day 11, then sacrificed at day 12 F) serum ferritin ELISA at day 12, black dashed line: normal range, red dashed line: abnormal or pharmacologically lowered ferritin. G, H) Effect of DFO treatment on tumor growth in the metastatic Bo1 model G) treatment diagram: mice were inoculated by left ventricle injection, then imaged by BLI to establish tumor engraftment at day 4 and growth at day 8 and 11, treated with 4 injections of DFO 200mg/kg over 5 days, and intravenous AF750-Tf on day 10, H) total-body BLI at 4, 8, and 11 days after intracardiac inoculation of Bo1, average radiance in p/s/cm^2^/sr (N = 5/group).

MCF-7 intraductal breast cancer cells were treated for 24h with ammonium citrate (FAC), deferoxamine (DFO), or vehicle, then incubated with AF488-Tf for 30 minutes. Live optical microscopy showed some cytoplasmic staining in control cells, little to no staining in FAC-treated cells, and enhanced cytoplasmic and membrane staining in DFO-treated cells (Fig 5A).

To verify intracellular delivery, TNBC MDA-MB-231 cells were treated with DFO or vehicle then incubated with the pH-sensitive pHrodo™ Red transferrin, which becomes brightly fluorescent in the acidic environment of the endosome. Live microscopy showed diffuse and punctate intracellular fluorescence after tracer incubation, with higher intensity in DFO-treated cells (Fig 5B).

Microplate fluorimetry further showed that uptake of AF488-Tf in MDA-MB-231 was decreased by 2/3 upon FAC supplementation (P<0.001) and increased by 2/3 in cells pre-treated with DFO (P<0.05, Fig 5C). Luminal A breast cancer cells T47D showed no decrease in uptake of AF488-Tf upon iron supplementation (FAC 100-200μM, +1% to -23%). However, DFO increased transferrin uptake by 40% (P<0.01) at 80 μM DFO (not shown), by 75% at 100μM (P<0.01, not shown), and doubled it at 200 μM (P<0.0001, Fig 5D). In murine luminal B cells Bo1, treatment with FAC for 48h did not decrease uptake of AF488-Tf, but 200 μM DFO increased transferrin uptake by 130% (P<0.001, Fig 5E).

These results demonstrate that iron chelation can enhance uptake of transferrin by breast cancer cells.

### 3.7 Short-term iron chelation in the C57Bl/6 breast cancer metastasis Bo1 model

In order to translate the use of DFO to improve delivery of transferrin conjugates to breast cancer in vivo, two potential limitations needed to be addressed: safety of the treatment, and effects on tumor growth that may add confounding factors.

Tolerability of DFO in combination with labeled transferrin was tested in non-tumor bearing mice, receiving six intraperitoneal injections of DFO 100mg/kg or 200mg/kg over 11 days, then one intravenous injection of Tf-750 (Fig 5E). No behavioral changes, alterations in fur or posture, or macroscopic anomalies at necropsy were noted for either dose of DFO. Further, body weight remained stable (S5D Fig).

In order to test whether chelation had caused iron deficiency, we measured ferritin levels in the serum by ELISA. Long-term DFO treatment was previously reported to induce 20–30% decrease in serum ferritin in mice [78, 79]. However, all of our mice showed ferritinemia in the range reported for age- and strain- matched healthy mice [80], with no differences between treatments (Fig 5F).

DFO was previously reported to decrease growth of breast cancer in vitro and in primary tumors [81, 82]. To determine whether DFO treatment would affect the growth in the luminal B metastasis model, 6 week old C57Bl/6 were injected with Bo1 breast cancer cells in the left ventricle, imaged for engraftment on day 4, treated, and serially imaged by BLI (IVIS)(Fig 5G). DFO (200mg/kg i.p., N = 5) or vehicle (HBSS, N = 5) were administered on days 7, 8, 10 and 11. No weight loss (S5D Fig) or macroscopic signs of toxicity were observed in any of the groups. Serial ventral total body BLI showed constant increase of luminescence, with no differences in tumor growth between vehicle- and DFO-treated mice (Fig 5H).

### 3.8 Iron chelation improves the localization of fluorescently labeled transferrin to tumor sites in the Bo1 metastatic luminal B breast cancer model

**Fig 6:**
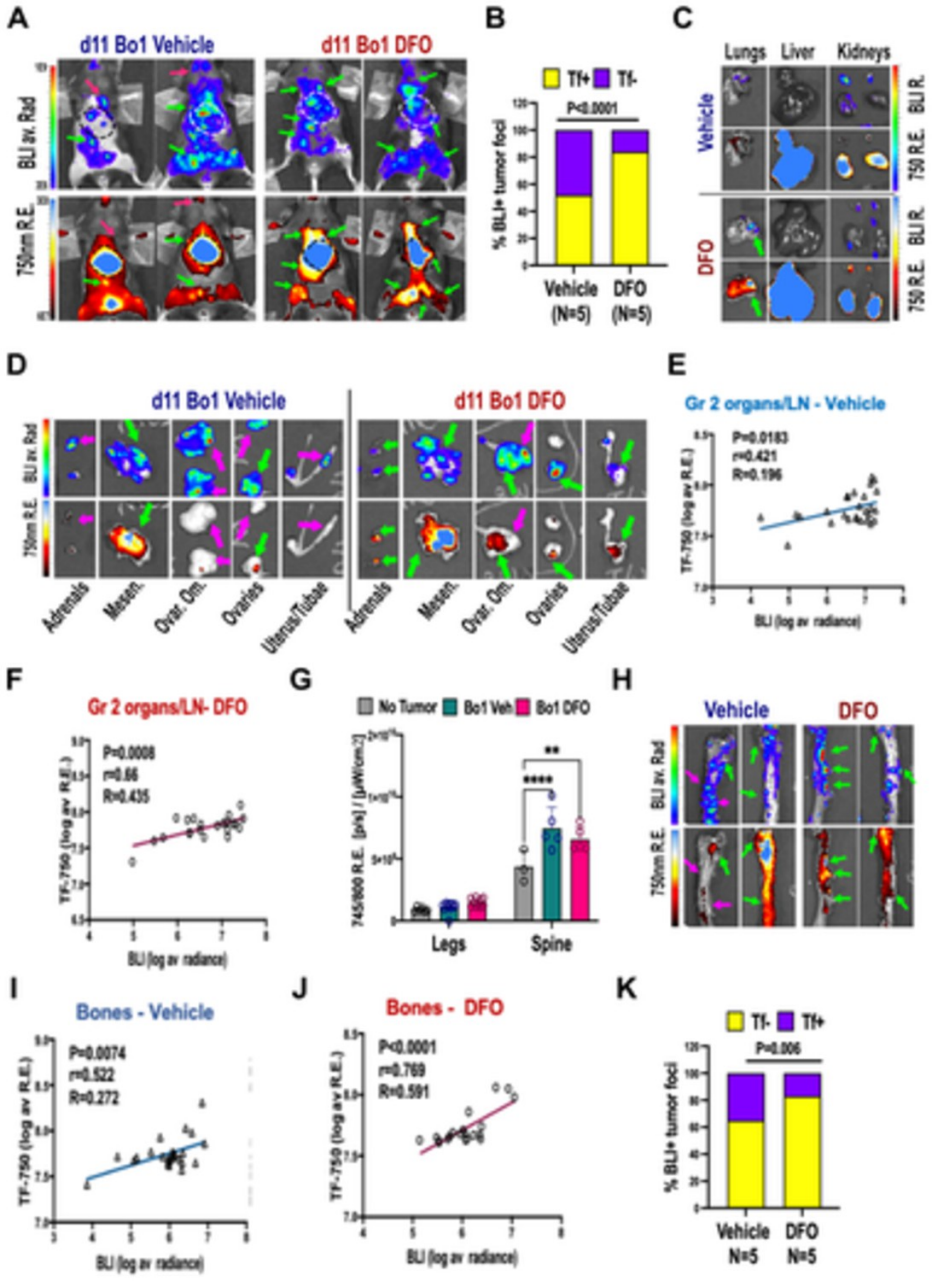
Iron chelation in metastatic Bo1 enhances the uptake of transferrin (AF750) at tumor sites. A-B) In vivo imaging with BLI and AF750 A) representative images of two tumor-bearing mice treated with vehicle (left) and two with 4 i.p. injections of DFO 200mg/kg (right), by BLI (top, rainbow pseudocolor of average radiance) and 750 nm epifluorescence (bottom, blue-hot psuedocolor of average radiant efficiency), red arrows: tumor lesions seen by BLI but not AF750, geen arrows: tumor sites where BLI and AF750-Tf co-localize, B) percentage of tumor foci by BLI that did (yellow) or did not (purple) show AF750-Tf accumulation in mice treated with vehicle versus DFO, P<0.0001 by Fisher’s exact test. C-J) ex-vivo imaging by BLI and AF750, representative images of BLI (top) and 750nm epifluorescence (bottom) (C, G, H), correlation between BLI and AF750 (E,F, I, J), bar graphs (G,K), C) example of group 1 organs ex-vivo imaging in vehicle (top) and DFO—treated (bottom) mice; D) representative image of adrenal glands, mesenter (fat, vessels, and lymph nodes), ovarian omentum, ovaries, uterus and tubae in vehicle (left) and DFO -treated (right) mice; E-F) correlation between tumor burden and AF750-accumulation in ROI from group 2: X axis, Log-transformed average radiance (photons/s/cm^2^/sr), Y axis, Log-transformed average radiant efficiency ((photons/s/cm^2^/sr)/(μW/cm^2^) E) vehicle-treated mice and F) DFO-treated mice; G) comparison of AF750-Tf uptake in the legs and spines of non tumor-bearing (N = 3, grey bars, No Tumor), Bo1 mice injected with vehicle (N = 5, teal), and Bo1 mice treated with DFO (N = 5, red); **P<0.01, ****P<0.0001 by two-way ANOVA and Šidák test; H) representative images of spines from two vehicle-treated (left) and two DFO-treated (right) BO1 mice I-J) correlation between tumor burden and accumulation of Tf in the bones (pelvis, legs, spine) of I) vehicle-treated (P = 0.0074, Pearson’s r = 0.522, R = 0.272) and J) DFO-treated (P<0.0001, r = 0.769, R = 0.591); K) percentage of tumor foci by ex-vivo BLI that did (yellow) or did not (purple) show AF750-Tf accumulation in mice treated with vehicle versus DFO, P = 0.006 by Fisher’s exact test.

Having established a high-load disseminated tumor burden in Bo1-injected mice with no interference from treatment (Fig 5F–5H), dual in vivo imaging was performed on day 11, 24h after injection of AF750-Tf (Fig 6A). Luciferase activity was evident in multiple sites in tumor-bearing mice. Fluorescence imaging showed accumulation of AF750 in the liver (as previously, Fig 4) and at several foci throughout the body.

To evaluate whether DFO had improved uptake of AF750-Tf, BLI and epifluorescence sequences were compared, counting the number of foci that were caught in both modalities versus only by BLI (Fig 6B). DFO-treated mice had a significantly higher percentage of metastatic foci accumulating AF750-Tf (84% ± 28% vs 52%±2.8%, Fisher’s test P<0.0001).

Dual ex-vivo imaging demonstrated broad tumor involvement of several organs and tissues, consistent with lymph node, peritoneal, bone, and organ metastases. To investigate the ability of transferrin conjugates to target metastases, we investigated the qualitative and semi-quantitative association between regions of high intensity of epifluorescence and BLI, and correlated the radiance and radiant efficiency for epifluorescence in fixed-shape ROI as previously described [27]. Based on previous experiments on Tf uptake in non-tumor mice (Figs 4 and S4), target tissues and organs were divided into three groups: high background (group 1, Fig 6C), soft tissue sites of metastases (group 2, Fig 6D), and bones (group 3).

Organs in group 1 had high fluorescence in all mice, consistent with high physiological uptake or involvement in clearance of AF750-Tf, with no difference between groups (Figs 6C and S6A ). Occasional redistribution of fluorescent signal within the organ towards tumor-affected areas, could be observed in the lungs (Fig 6C arrow, S6B Fig). However, overall no correlation was found between fluorescence and luminescence in either vehicle- or DFO-treated mice (S6C and S6D Fig).

Group 2 included organs with lower background where most metastatic foci were visible, namely mesentery, ovaries, uterus and fallopian tubes, ovarian omentum, and adrenal glands (Fig 6D). Vehicle-treated mice showed some correlation between BLI and epifluorescence in this group of organs (P<0.05, r = 0.42, Fig 6E). In DFO-treated mice, however, the correlation between tumor load and AF750-Tf uptake was significantly improved (P<0.001, r = 0.66, Fig 6F).

Similar to the 4T1/BalbC model, vertebral metastases increased AF750-Tf uptake relative to non-tumor mice in the spine of both vehicle- and DFO- treated Bo1 d11 mice (Fig 6F and 6G, P<0.001 S6E Fig). Bone metastases to the hindlimbs and pelvis in this cohort were generally small, and total radiant efficiency was non superior to non-tumor controls, similarly to what observed in 4T1 mice. However, we found a positive correlation between luciferase activity and AT750-Tf uptake in vehicle-treated mice (P<0.01, r = 52, Fig 6H) and a very strong correlation mice treated with DFO (p<0.0001, r = 0.77, Fig 6I).

Ultimately aiming at assessing whether transferrin conjugates would target a metastatic focus, we counted the tumor foci as seen by ex-vivo BLI that also showed AF750-Tf accumulation. Vehicle-treated mice showed AF750-Tf signal on average in 64.6% (SD 18.7%) of BLI-positive foci, while DFO-treated mice had detectable uptake of TF750 in 83.2% of ROI (SD 13.2%). Contingency analysis by Fisher’s exact tests suggested a significant difference in favor of DFO-treated mice (P = 0.015, Fig 6E).

Having observed that Tf-conjugated fluorophores were hardest to trace in smaller tumor lesions (Figs 4 and 6), we set out to test the effects of DFO at an earlier stage of the diffuse Bo1 tumor model, characterized by significantly lower tumor burden.

**Fig 7:**
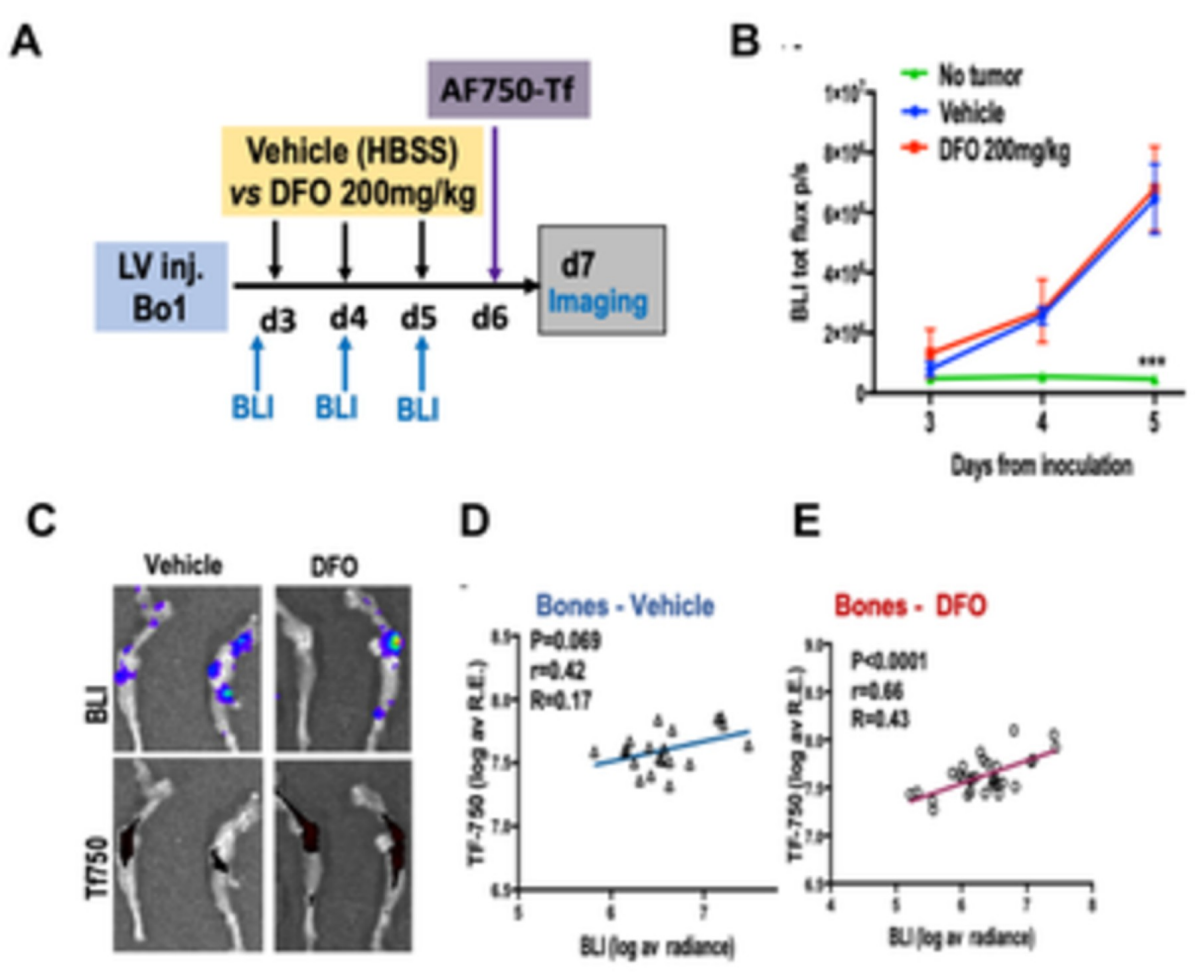
Iron chelation enhances the uptake of transferrin (AF750) in low-tumor burden Bo1 model. A) Experimental design: mice were inoculated with Bo1 i.c. and treated from day 3 imaged with vehicle (V) or DFO; injection of AF750-Tf on day6 and imaging day 7; B) quantification of total-body radiance over time show no difference in tumor bearing mice treated with vehicle or DFO, *** P<0.001 in non tumor-bearing controls C) ex-vivo imaging of bones from one vehicle and one DFO-treated mice by BLI (top) and AF750-tf epifluorescence (bottom), D-E) Correlation between BLI and AF750-Tf fluorescence in the bones (legs, pelvis, spine) of D) vehicle-treated mice (P = 0.069, r = 0.42, R = 0.17, N = 5) and E) DFO-treated (P<0.0001, r = 0.66, R = 0.43, N = 5) mice.

Female C57Bl/6 mice were injected Bo1 cells in the left ventricle, and BLI was performed on day 3 to establish tumor engraftment before treatment (Fig 7A). Mice received daily i.p. injections from day 3 to day 5 with DFO 200mg/kg or HBSS (vehicle), then intravenous AF750-Tf on day 6, with no weight loss or signs of toxicity (S7B Fig). Serial imaging with BLI to monitor tumor growth showed no difference between DFO-treated and untreated mice (Figs 7B and S7A), despite the high dose and early treatment initiation.

Ex vivo BLI showed few and small metastases at several sites. In visceral organs and soft tissues, no correlation between BLI and AF750-Tf fluorescence was found in vehicle- (P = 0.108, r = 0.277, R = 0.077, S7C Fig) nor DFO-treated animals (P = 0.067, r = 0.250, R = 0.064, S7D Fig). In the leg bones and spine few and small metastatic foci could be identified by BLI (Fig 7C) independent of treatment. In vehicle-treated mice, unlike in the late-stage experiment, no significant correlation was found between BLI and (P = 0.069, r = 0.42, R = 0.17) (Fig 7C). By contrast, in DFO-treated mice BLI radiance and epifluorescence from AF750-Tf were strongly correlated (p<0.0001. r = 0.66, R = 0.43).

Overall, this data provides proof-of-principle that systemic iron chelation can increase Tf uptake in breast cancer metastases.

## 4. Discussion

In this study, we set out to characterize gene and protein expression of transferrin receptor (TfR, *TFRC*) of breast cancer broadly and systematically. TfR was expressed in breast cancer, including untreated tumors, residual tumor tissue after neoadjuvant chemotherapy, and metastases. Preclinical models showed variable uptake of fluorescently-labeled transferrin at metastatic sites. Treatment with DFO [30, 76] increased the uptake of transferrin conjugates by breast cancer cells in vitro and in a mouse model of metastatic breast cancer.

Iron is essential to cellular metabolism, growth, and signaling [82, 83], and its uptake depends on transferrin receptors, primarily TfR (*TFRC*) [6, 16, 84]. TfR is present in all cells, but cancer cells overexpress it in order to increase iron uptake and sustain higher metabolism and proliferation [7, 16, 19–22] (S1 Table and Figs 1D and S1A and S1B). Because of this, many groups have developed diagnostic and therapeutic agents by binding to transferrin drugs or imaging agents to target cancer cells [5, 7–12, 22, 23, 25, 26, 28, 83, 85–87].

In breast cancer, biomaterials targeting transferrin receptor were tested in primary orthotopic tumors [5, 8, 9, 25–28, 86, 87], where a 70% average prevalence of TfR expression is well established [20, 21, 63]. For primary breast cancer, however, effective treatments are available. Innovative therapies are needed for metastatic or previously treated cancers, which respond poorly to chemotherapy but are underrepresented in preclinical research. Our study therefore started by analyzing expression of TfR in all breast cancers, including post-chemotheraphy and metastatic tissue.

High *TFRC* expression was confirmed (Figs 1 and S1A and S1B), particularly in cases with high pathological grade [7, 20, 21, 62] (Fig 1C) and of triple negative subtype [21, 62, 64, 65, 86] [88] (Fig 1B). In line with the reported association with Ki67 (*MKI67*, S1C Fig) [8, 64, 89], *TFRC* correlated with a set of genes associated with highest proliferation in breast cancer [62] (S1C Fig). Negative correlation with ER [61, 63] and PR and lower expression in luminal subtypes were also confirmed for *TFRC* RNA. Immunohistochemistry (IHC) was also consistent with previous reports, showing an average of 70% (range 45–95%) TfR-high staining across all tumor stages and subtypes. In fact, ER+ and slower growing luminal A tumors could express TfR at high levels; vice-versa, TNBC or high-grade tumors could show weak TfR staining.

Previous studies reported that transferrin overexpression in the primary tumor at diagnosis predicted decreased response to drugs [62], and actively promote the development of metastases [66]. Here, we investigated the expression of TfR in the tissue of breast cancer *after* neoadjuvant therapy and once metastases had already occurred.

Gene expression and IHC of breast cancer tissue showed no significant differences in TfR expression in patients treated with most chemotherapeutics. An exception were regimens based on trastuzumab [39, 45, 70], which inhibits signaling upstream of overexpression [17] and decreased *TFRC* expression in all post-NAC tumors (Figs 2A and S2I). No association was found between TfR expression and previous treatments with other regimens (Figs 2 and S2 and S3).

Despite being the major determinant of mortality in breast cancer, metastases are generally underrepresented in studies of patient samples. In a custom-made TMA with matched samples from primary tumor vs bone metastases IHC showed that most patients with TfR-positive primary tumors retained expression of TfR in bone metastases, and 63% of tumors that were negative would express TfR in the metastatic site (Fig 3).

Similar to patients, preclinical models showed generally high but variable TfR expression in primary and metastatic sites. Uptake of fluorescently-labeled transferrin was evaluated in mice with disseminated 4T1 or Bo1 tumors (Figs 4–7). By ex vivo imaging, uptake of Tf was significantly higher in the spine of tumor bearing mice than controls. In other sites of metastases, uptake could be very variable or no higher than non-tumor.

Of note, in metastatic models, tumor foci are often multiple, usually smaller, and intrinsically more variable within or across individuals (S4G-S4I Fig). More to the point, while in primary tumors cancer cells constitute 50% or more of a well-defined region, metastases are surrounded by the non-tumor cells of the native tissue. For molecular imaging, such as 45-Ti transferrin PET [10], this challenges both elements of the signal to noise ratio (SNR). Fewer cancer cells grouped in a sub-millimetric metastasis collectively take up less tracer (low S) than a large tumor (high S). As the “noise” depends on uptake from the surrounding tissue, organs with physiologically high uptake of transferrin (e.g. the liver) will have higher noise than tissues with different functions (e.g. non-tumor fat pads) (Figs 4 and S4E and S6A and S6B). Overall, metastatic models are therefore a stricter challenge than the initial tests on primary tumors. In this study, the performance of tumor Tf- imaging in this more stringent test motivated the search for a way to improve the uptake of transferrin conjugates by cancer cells.

Breast cancer cells upregulate TfR through both oncogenic [8, 17] and iron regulatory signaling networks [15, 17, 18]. In vitro, loss of TfR function, Tf starvation, or long-term potent iron chelation inhibit growth and viability of breast cancer cells [7, 19, 30, 82, 83, 90]. The adaptive response to iron scarcity is the overexpression of TfR, observed in breast cancer experiments [76, 77], and previously used to increase the uptake of transferrin conjugates in vitro in glioma or leukemia cells [22, 85, 91]. Here, a non-lethal treatment with DFO significantly increased binding and endocytosis of fluorescently-labeled transferrin by luminal A, luminal B, or TNBC cells in vitro (Fig 5A–5C).

In vivo, studies of primary breast cancer showed that long-term treatment with DFO inhibitors reduced tumor growth [29, 31, 81, 91]. In the Bo1 model of breast cancer metastases, short-term treatment with DFO did not affect tumor growth, even when administered early after engraftment (Fig 7). This could be due to the prevalence of the microenvironment [92], where myeloid cells can secrete transferrin supporting metastatic growth [93], or to overall lower dosing. In both patients [94] and mice [78, 79] long-term high-dose DFO can strain systemic iron metabolism, reflected in reduced ferritin levels. In our short treatment regimen, however, mouse ferritin levels remained normal (Fig 5F). This suggests that short-term DFO treatment may be able to elicit adaptive upregulation of TfR without inducing cytoreductive nor potentially toxic iron starvation. In the Bo1 metastatic model, *in vivo* and *ex vivo* imaging showed that short-term DFO treatment improved uptake of fluorescently-labeled transferrin at tumor sites. DFO-treated mice showed stronger correlation between tumor burden and AF570-Tf relative to vehicle treated controls (Figs 6 and 7, S6 and S7). Further, more metastatic foci accumulated labeled transferrin in DFO- versus vehicle-treated mice (Figs 6 and 7). These findings suggest that treatment with DFO could improve localization of TfR-targeted biomaterials to metastatic breast cancer without disrupting systemic iron homeostasis.

Preclinical studies in breast cancer showed potential effects of iron chelators in reducing tumor growth [30, 31, 33, 77, 81] or enhancing chemotherapy [18, 31, 90]. Clinical studies are currently ongoing in breast cancer (e.g.NCT05300958, NCT04435028) and other tumors (NCT05184816, NCT03652467). However, encouraging safety data derives from chelation in other non-iron overload conditions [95–99], and other solid tumors [100, 101]. In thsese, reactions to DFO were mostly low-grade toxicity, such as irritation at site of injection and anorexia in up to 25% of patients [97, 98, 101, 102]. Grade 2–3 adverse events were rare and reversible at discontinuation of DFO treatment [97, 101], and no grade 4 adverse events were reported [97, 98, 101, 102]. Deferiprone [95, 96, 99] and deferasirox [103] are iron chelators that are orally available. In conditions without iron overload, they are reported to be more potent and convenient, but associated with greater side-effects, including QT-prolongation [99], reversible renal [103] or hepatic dysfunction [99], and neutropenia up to grade 4 agranulochytosis [95, 96, 99, 103]. While no patients developed anemia or sideropenia, all patients treated with iron chelators showed decrease in ferritinemia over months of treatment. In our experiments, short-term DFO did not affect serum ferritin levels. While cytoreductive protocols may require more potent and higher dose chelators, this data suggests that short-term administration of DFO to enhance the uptake of transferrin conjugates may be safe and well tolerated.

Transferrin targeting for breast cancer has been developed into promising technologies [5, 7–12, 22, 23, 25, 26, 28, 83, 85–87]. By showing overexpression of transferrin receptor in metastatic and pre-treated breast tumors from a large population, our study expands on the potential indications of transferrin conjugates for drug delivery or imaging. At the same time, we show that in individual tumors and cells, expression of the receptor and uptake of Tf-conjugates can be variable, and subject to changes. This suggests that TfR-directed nanotechnologies should follow a theranostic approach, whereby binding of the homing ligand is verified by imaging prior to or while administering targeted treatments. Further, combination with FDA-approved iron chelators may improve delivery of TfR-targeting agents to breast cancer metastases, potentially improving specificity of delivery and efficacy of treatments. Studies are currently underway to evaluate therapeutic uses of TfR-targeting, enhanced by iron chelation, of breast cancer and the macrophages in the tumor microenvironment, and their potential use to sensitize breast cancer to chemo- and immunotherapy.

## 5. Conclusions

A wide survey shows high expression of transferrin receptor not only in primary breast tumors, but also in refractory/relapsing breast cancer and its visceral and bone metastases. In these severe cases, molecular targeting through transferrin receptor could be a promising strategy to develop efficacious treatments to improve survival. *Due to variability of expression*, *combinations with molecular imaging or use of theranostics may be necessary to identify patients with highest target expression*. Systemic manipulation of iron availability through non-toxic chelators could increase transferrin uptake by cancer cells, boosting the efficacy of targeted therapies.

## Supporting information

S1 Table Cancer vs non-tumor tissue expression of *TFRC* from the GENT2 database; P value for T-test of cancer vs non-cancer and Log 2 of fold-change (Log2 FC). (PDF)

S1 Fig A, B) Expression of TFRC in breast cancer versus normal tissue in A) the TGCA dataset (N = 6041) and B) GENT2 dataset (N = 1211), C) Correlation matrix of expression level (TPM) of TFRC with proliferation-associated genes (MKI67, CCNB1, CCNA2, CDK2, CDK4, CDK6, TOP2A, TOP1), oncogenes (MYC, NRAS, KRAS), receptors (ESR1, ESR2, PGR, ERBB2/Her2) and iron regulatory IREB2 in breast cancer (N = 1097 tumors from TGCA), color scale/cell numbers: Pearson’s correlation r. D-F) Tumor microarray IHC for TfR H-scoring D) representative images of scores 0, 1, 2, 3, E, F) H-score of breast cancer samples based on TNM staging (total N = 87) E) by tumor score T, F) by lymph node score N.
(PNG)

S2 Fig A) TFRC expression in TCGA samples stratified by previous exposure to chemotherapy; B, C) Expression of CDKN1 (p21, affected by epirubicin and taxanes) in B) GSE21974 dataset of breast cancer biopsies f before (Pre) and after neoadjuvant chemotherapy with four cycles of epirubicin and cyclophosphamide prior to taxane (NAC;EC-T); C) in GSE18728 in the diagnostic biopsy (pre), in biopsy after one cycle (Cy Tx), and at tumor resection (Res) D, E) in dataset GSE28826 of 14 patient-matched samples of breast cancer before (pre) and after NAC with taxanes and antracyclins (NAC:AT) D) expression of CDKN1 (p21) and E) of TFRC; F, G) In GSE153470 of patients before (Pre) and after (Post-AI) endocrine therapy with aromatase inhibitors, expression of F) of CCND1 (affected by aromatase inhibitors) and G) TFRC; H) In GSE114082 expression of CCND1 in matched Her2+ breast cancer pre- and post- NAC with trastuzumab; I) In GSE130788 expression of TFRC in HER2+ breast tumors (N = 99 samples) at baseline versus after six cycles of NAC with taxane, carboplatin, and Her2 inhibitors: TCTy (docetaxel, carboplatin, Lapatinib, N = 29 patients), TCHTy (docetaxel, carboplatin, trastuzumab, lapatinib, N = 38), TCH (docetaxel, carboplatin, trastuzumab, N = 31). J) waterfall plot of H-score in matched TNBC biopsies before versus after NAC (N = 6 patients).
(PNG)

S3 Fig A) WHIM69 were treated with paclitaxel (30mg/kg) and carboplatin (50mg/kg) (Carbotaxol) versus vehicle on days 44,51,42 from implant; vehicle-treated tumors were harvested on day 62, NAC on day 111, and relapse 119 days later; treatment scheme, representative images of TfR staining and quantification scatter plot post-vehicle, post-NAC, and at relapse; B) Tfrc expression in GSE165393 set of spontaneous metastases of the MMTV-PYMT breast cancer model (N = 2); C) GSE62598 expression of tfrc in 4T1 murine TNBC isolated cells, orthotopic primary tumor, or metastases to bone, liver, or lung (N = 3); D) IHC scoring for Bo1 tumors from injection in the mammary fat pad (MFP, N = 2) or growing after left-ventricle injection as metastasis to the kidney, lung, or bone; E, F) TfR IHC of 4T1 MFP tumors (N = 4), and intracardiac injection-induced metastases to the kidneys (N = 1), lungs (N = 6), or bones (N = 2) as E) representative images, F) TfR H-Score.
(PNG)

S4 Fig A) Representative images of in vivo and ex-vivo epifluorescence imaging of non-tumor bearing C57Bl/6 before, 10 minutes, 6h, and 24h post injection of AF750-Tf (left) and liver, kidneys, lungs, and legs of mice 24h from injection of vehicle (PBS, left) versus AF750-Tf (right); B-D) gene expression of Tfrc (teal) and Tfr2 (plum) in three independent normal mouse transcriptome on gene expression Atlas; E) correlation between organ expression of Tfrc and Tfr2 and uptake of AF750-Tf in the same organ; x axis: sum of the average expression of Tfrc and Tfr2 in TPM; y axis: logarithm of the difference between average radiant efficiency in the organ ROI and in the background (ΔAF750-Tf av) in healthy mice 24h after ATF750-Tf injection. F-I) in 4T1 tumor bearing mice (4T1 i.c. day 12) versus control mice (No Tumor) injected with AF680-Tf, F) average radiance of in vivo BLI at day 12 in ventral and dorsal positions, G-H) total radiance in ex-vivo BLI in lungs, kidneys, legs, and spine of mice sacrificed (G) 6h and (H) 24h from AF680-Tf injection, I) comparison of ex-vivo AF680-Tf and BLI images of leg bones.
(PNG)

S5 Fig A) live cell optical microscopy showing uptake of AF488-Tf in MCF7 cells in normal growing conditions, exposed to FAC 100μM, or DFO 100μM; blue Hoechst, green AF488-Tf; B, C) AF488-Tf uptake by T47D cells pre- treated 24h; B) 80 μM DFO vs. vehicle, C) 100 μM DFO or μM FAC vs vehicle; 488/Hoechst fluorescence intensity. D) maintenance of body weight in non tumor-bearing mice treated with six injections of HBSS (vehicle, black), DFO 100mg/kg (teal), or DFO 200mg/kg (red). E) body weight at day 11 relative to day of intracardiac tumor inoculation of Bo1 mice treated with vehicle (teal), or Bo1 mice treated with DFO 200mg/kg (red), or non-tumor bearing controls (grey); black line 100% (no change), red line critical weight loss of 20% (none recorded in this study).
(PNG)

S6 Fig A) AF750-Tf uptake in the liver by total radiant efficiency in non-tumor (grey), day 11 Bo1 tumor bearing vehicle-treated (teal) or Bo1 DFO-treated (red) mice in group 1 organs lungs, liver, and kidneys B) representative examples of BLI (top, radiance in rainbow pseudocolor scale) and AF750-Tf imaging (bottom, radiant efficiency in blue hot scale) of lungs from non tumor bearing (No Tumor, left), day 11 Bo1 tumor bearing mice treated with vehicle (Bo1 Vehicle, central), and day 11 Bo1 mice treated with DFO (Bo1 DFO, right); C, D) correlation between correlation between BLI (log of average radiance) and AF750-Tf (log average radiance efficiency) in rectangular ROI on soft tissues/organs with low incidence of metastases and/or high basal uptake in Bo1 mice treated with vehicle (left) or DFO (right); D) comparison of ex-vivo BLI and AF750-Tf imaging in three representative Bo1 mice per treatment (medium, non detectable, high) treated with vehicle (left) or DFO (right); E) AF750-Tf uptake in the leg bones by total radiant efficiency in day 11 Bo1 mice treated with vehicle (teal) or DFO (red) versus non-tumor controls (grey). ***P<0.001 by one-way ANOVA Tukey post-hoc test.
(PNG)

S7 Fig A) In vivo BLI representative image (rainbow scale for radiance) at day 3 and day 5; B) maintenance of mouse weight in Bo1-injected mice treated with vehicle (N = 4, teal) or DFO (N = 6, red); C) correlation between log (average radiance) from luciferase and log (average radiance efficiency) for Tf750 in rectangular ROI on soft tissues/ visceral organs at day 6 post- Bo1 inoculation.
(PNG)

## List of abbreviations

AF750: Tf AlexaFluor750-conjugated transferrin
ANOVA: analysis of variance
BAT: brown adipose tissue
BLI: bioluminescence imaging
DFO: deferoxamine
ER: estrogen receptor
FAC: ferric ammonium citrate
FDA: Food and drug administration
FMT: fluorescence molecular tomography
FWA: Federal Wide Assurance
GENT2: Gene expression database of Normal and Tumor tissues
GEO: gene expression omnibus
GFP: green fluorescent protein
h: hours
HER2: human epithelial growth factor receptor 2
IHC: immunohistochemistry
IRP1: Iron responsive protein 1
IRP2: Iron responsive protein 2
LN: lymphnode
Luc: luciferase
MFP: mammary fat pad
NAC: neoadjuvant chemotherapy
NEAA: non essential amminoacids
OD: optical density
PDX: patient derived xenografts
PerL: persistent luminescence imaging
PR: progesterone receptor
R-LN: regional lymph node
RNAseq: RNA sequencing
ROI: region of interest
RT: room temperature
scRNAseq: single cell RNA sequencing
SD: standard deviation
SEM: standard error of the mea
TCGA: Cancer Genome Atlas
Tf: transferrin
Tf-488: AlexaFluor 488-conjugated human transferrin
TfR: transferrin receptor
TFR2: transferrin receptor 2
TMA: tumor microarray
TNBC: triple negative breast cancer
TPM: transcripts per million
WAT: white adipose tissue
