# Supplementary material for: Transferrin receptor in primary and metastatic breast cancer: Evaluation of expression and experimental modulation to improve molecular targeting
Source: PLoS One. 2023 Dec 20;18(12):e0293700. doi: 10.1371/journal.pone.0293700 (PMC10732420; doi:10.1371/journal.pone.0293700)
Supplement: S1 Table — (PDF) [file pone.0293700.s001.pdf]

Classification	Group	N. Samples	Av FPKM-UQ+1	St Dev	ANOVA
T	T1	310	11.2	0.9	*ns*
	T2	705	11.3	1.1	*ns*
	T3	150	11.1	1.1	*ns*
	T4	53	11.4	1	*ns*
N	N0	561	11.3	1.03	*ns*
	N1	416	11.3	1.03	*ns*
	N2	132	11.5	1.07	*ns*
	N3	83	11.2	1.14	*ns*
M	M0	1021	11.3	1	*ns*
	M1	24	11.3	1.3	*ns*

Supplementary table 1. Data on expression of TFRC in breast cancer from the TGCA database, stratified according to the TNM system. Ns non significant
