# Supplementary figures and images for: Transferrin receptor in primary and metastatic breast cancer: Evaluation of expression and experimental modulation to improve molecular targeting
Source: PLoS One. 2023 Dec 20;18(12):e0293700. doi: 10.1371/journal.pone.0293700 (PMC10732420; doi:10.1371/journal.pone.0293700)

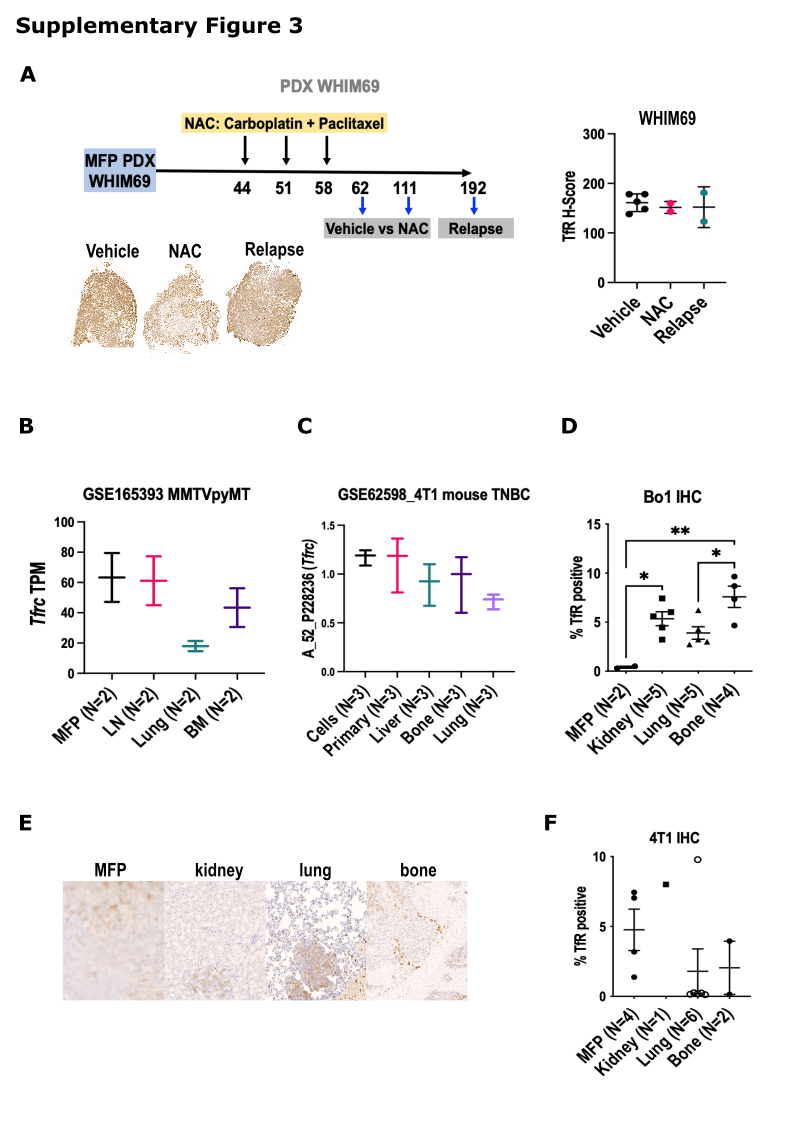

Supplement: S3 Fig — A) WHIM69 were treated with paclitaxel (30mg/kg) and carboplatin (50mg/kg) (Carbotaxol) versus vehicle on days 44,51,42 from implant; vehicle-treated tumors were harvested on day 62, NAC on day 111, and relapse 119 days later; treatment scheme, representative images of TfR staining and quantification scatter plot post-vehicle, post-NAC, and at relapse; B) Tfrc expression in GSE165393 set of spontaneous metastases of the MMTV-PYMT breast cancer model (N = 2); C) GSE62598 expression of tfrc in 4T1 murine TNBC isolated cells, orthotopic primary tumor, or metastases to bone, liver, or lung (N = 3); D) IHC scoring for Bo1 tumors from injection in the mammary fat pad (MFP, N = 2) or growing after left-ventricle injection as metastasis to the kidney, lung, or bone; E, F) TfR IHC of 4T1 MFP tumors (N = 4), and intracardiac injection-induced metastases to the kidneys (N = 1), lungs (N = 6), or bones (N = 2) as E) representative images, F) TfR H-Score. (PNG) [file pone.0293700.s004.png]

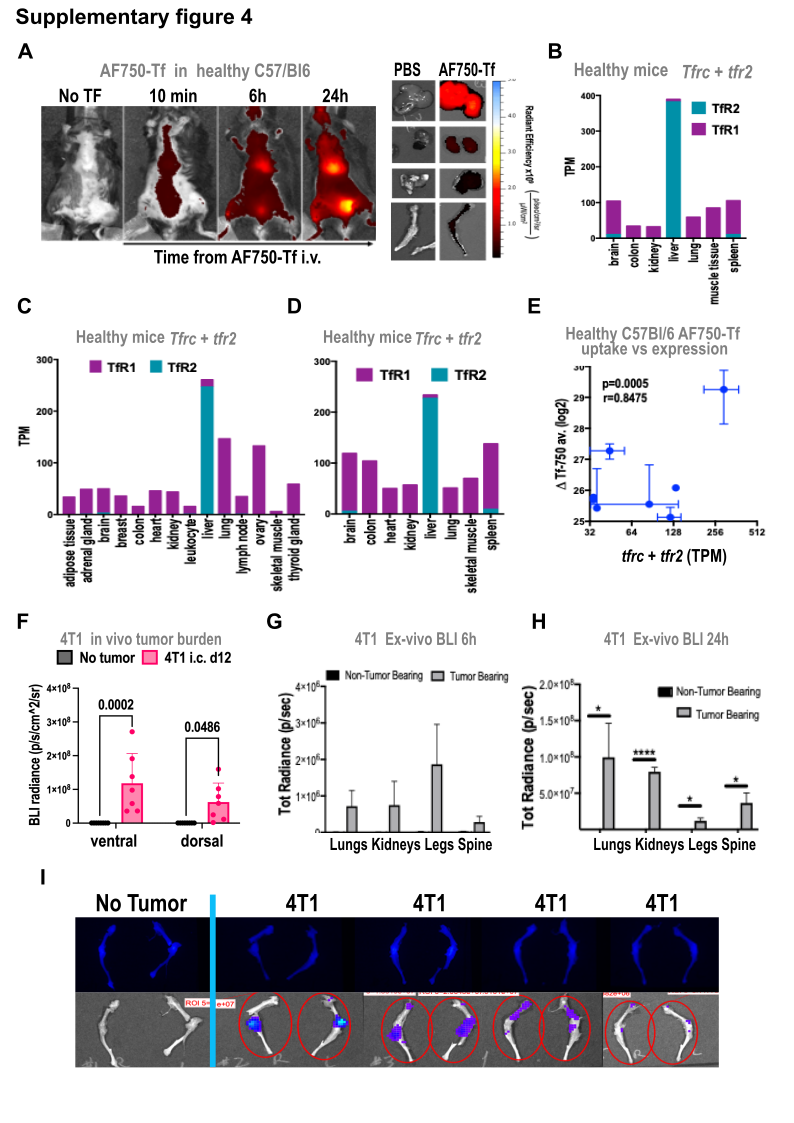

Supplement: S4 Fig — A) Representative images of in vivo and ex-vivo epifluorescence imaging of non-tumor bearing C57Bl/6 before, 10 minutes, 6h, and 24h post injection of AF750-Tf (left) and liver, kidneys, lungs, and legs of mice 24h from injection of vehicle (PBS, left) versus AF750-Tf (right); B-D) gene expression of Tfrc (teal) and Tfr2 (plum) in three independent normal mouse transcriptome on gene expression Atlas; E) correlation between organ expression of Tfrc and Tfr2 and uptake of AF750-Tf in the same organ; x axis: sum of the average expression of Tfrc and Tfr2 in TPM; y axis: logarithm of the difference between average radiant efficiency in the organ ROI and in the background (ΔAF750-Tf av) in healthy mice 24h after ATF750-Tf injection. F-I) in 4T1 tumor bearing mice (4T1 i.c. day 12) versus control mice (No Tumor) injected with AF680-Tf, F) average radiance of in vivo BLI at day 12 in ventral and dorsal positions, G-H) total radiance in ex-vivo BLI in lungs, kidneys, legs, and spine of mice sacrificed (G) 6h and (H) 24h from AF680-Tf injection, I) comparison of ex-vivo AF680-Tf and BLI images of leg bones. (PNG) [file pone.0293700.s005.png]

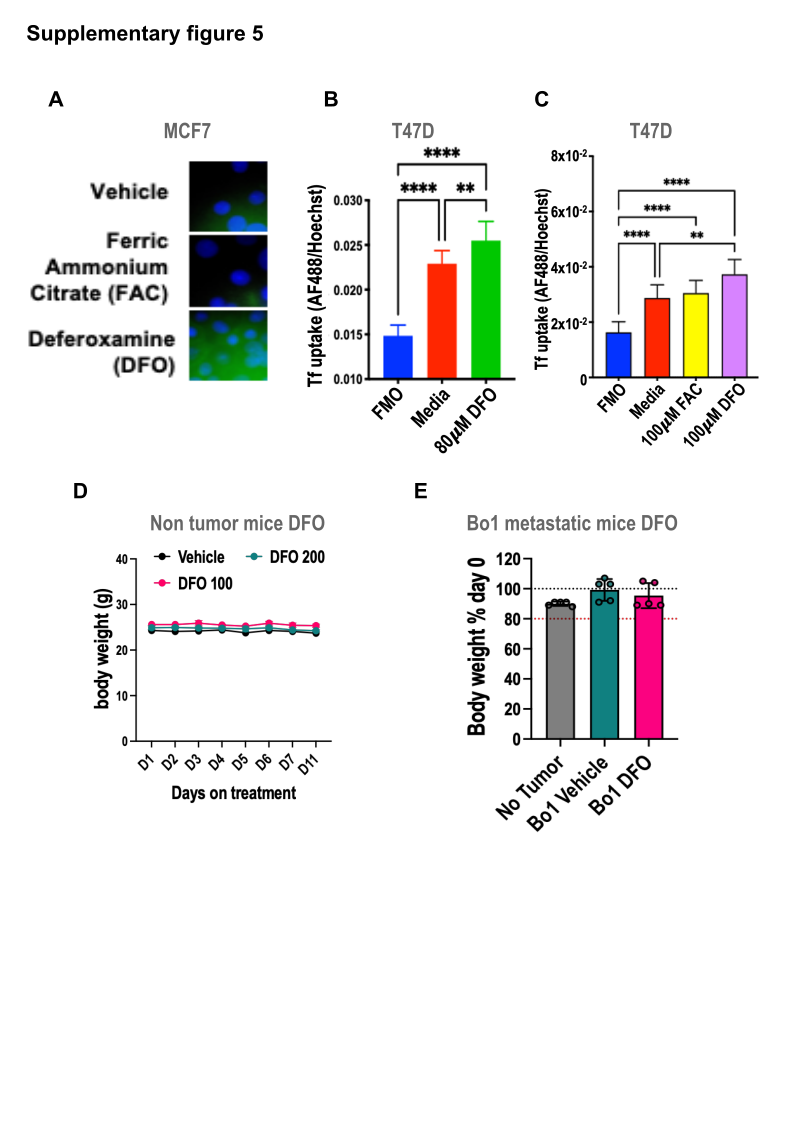

Supplement: S5 Fig — A) live cell optical microscopy showing uptake of AF488-Tf in MCF7 cells in normal growing conditions, exposed to FAC 100μM, or DFO 100μM; blue Hoechst, green AF488-Tf; B, C) AF488-Tf uptake by T47D cells pre- treated 24h; B) 80 μM DFO vs. vehicle, C) 100 μM DFO or μM FAC vs vehicle; 488/Hoechst fluorescence intensity. D) maintenance of body weight in non tumor-bearing mice treated with six injections of HBSS (vehicle, black), DFO 100mg/kg (teal), or DFO 200mg/kg (red). E) body weight at day 11 relative to day of intracardiac tumor inoculation of Bo1 mice treated with vehicle (teal), or Bo1 mice treated with DFO 200mg/kg (red), or non-tumor bearing controls (grey); black line 100% (no change), red line critical weight loss of 20% (none recorded in this study). (PNG) [file pone.0293700.s006.png]

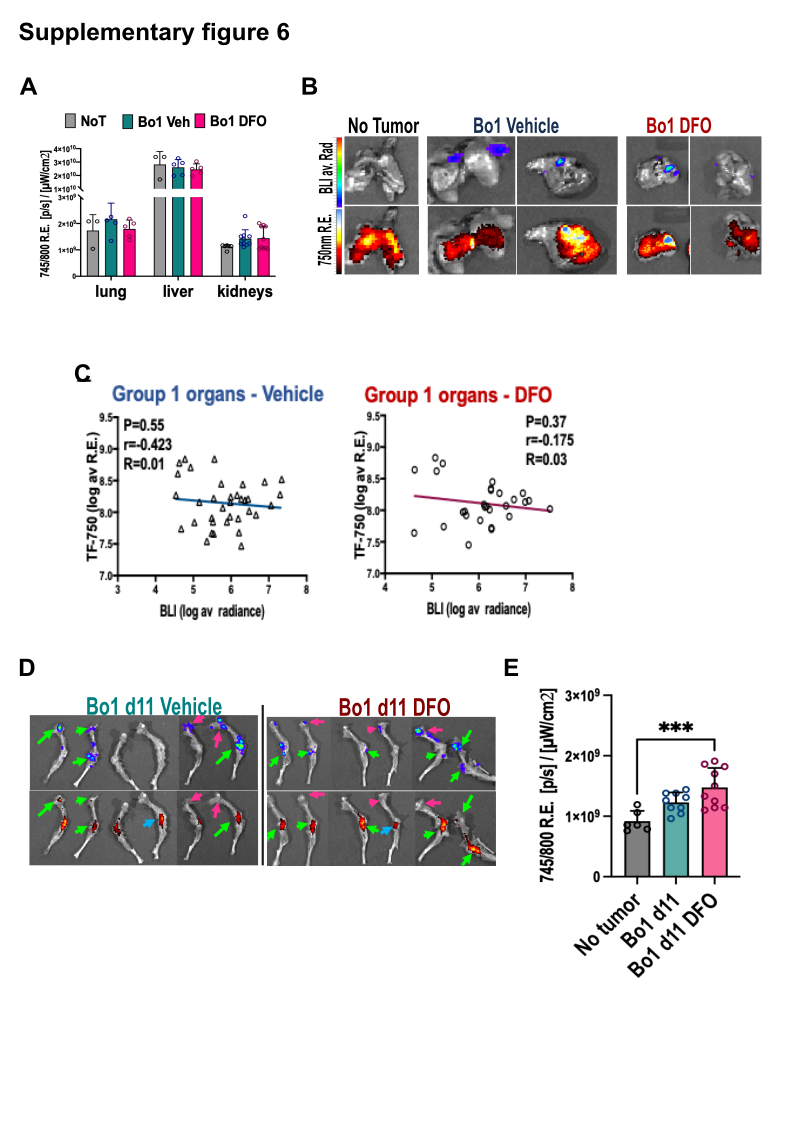

Supplement: S6 Fig — A) AF750-Tf uptake in the liver by total radiant efficiency in non-tumor (grey), day 11 Bo1 tumor bearing vehicle-treated (teal) or Bo1 DFO-treated (red) mice in group 1 organs lungs, liver, and kidneys B) representative examples of BLI (top, radiance in rainbow pseudocolor scale) and AF750-Tf imaging (bottom, radiant efficiency in blue hot scale) of lungs from non tumor bearing (No Tumor, left), day 11 Bo1 tumor bearing mice treated with vehicle (Bo1 Vehicle, central), and day 11 Bo1 mice treated with DFO (Bo1 DFO, right); C, D) correlation between correlation between BLI (log of average radiance) and AF750-Tf (log average radiance efficiency) in rectangular ROI on soft tissues/organs with low incidence of metastases and/or high basal uptake in Bo1 mice treated with vehicle (left) or DFO (right); D) comparison of ex-vivo BLI and AF750-Tf imaging in three representative Bo1 mice per treatment (medium, non detectable, high) treated with vehicle (left) or DFO (right); E) AF750-Tf uptake in the leg bones by total radiant efficiency in day 11 Bo1 mice treated with vehicle (teal) or DFO (red) versus non-tumor controls (grey). ***P<0.001 by one-way ANOVA Tukey post-hoc test. (PNG) [file pone.0293700.s007.png]

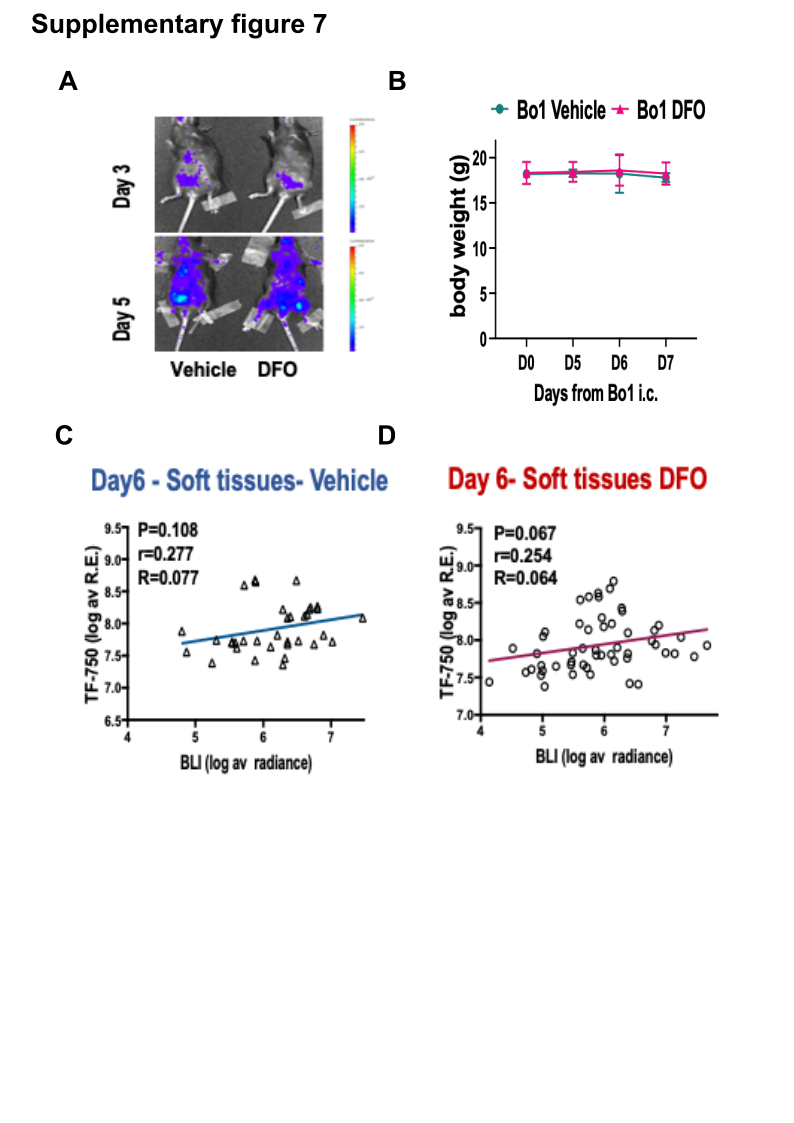

Supplement: S7 Fig — A) In vivo BLI representative image (rainbow scale for radiance) at day 3 and day 5; B) maintenance of mouse weight in Bo1-injected mice treated with vehicle (N = 4, teal) or DFO (N = 6, red); C) correlation between log (average radiance) from luciferase and log (average radiance efficiency) for Tf750 in rectangular ROI on soft tissues/ visceral organs at day 6 post- Bo1 inoculation. (PNG) [file pone.0293700.s008.png]
